# Total Syntheses and Anti-Inflammatory Studies of Three Natural Coumarins: Glycycoumarin, Glycyrin, and 3-*O*-Methylglycyrol

**DOI:** 10.3390/molecules29163942

**Published:** 2024-08-21

**Authors:** Ting Peng, Bin Long, Xiuli Yang, Na Wang, Ximeng Wang, Yujiao He, Hongbo Dong

**Affiliations:** 1 Anti-Infective Agent Creation Engineering Research Centre of Sichuan Province, School of Pharmacy, Chengdu University, Chengdu 610106, China; pengting221@cdu.edu.cn (T.P.); longbin8483@163.com (B.L.); 19861402559@163.com (N.W.); 13281217006@163.com (X.W.); 2 School of Clinical Medicine, Chengdu University of Traditional Chinese Medicine, Chengdu 610057, China; 18328729927@163.com

**Keywords:** natural products, licorice, coumarin, total synthesis, anti-inflammatory activity

## Abstract

Licorice (*Glycyrrhiza uralensis* Fisch), a significant traditional Chinese herbal medicine, has been extensively utilized in China to treat various ailments. Natural bioactive coumarins, glycycoumarin, glycyrin, and 3-*O*-methylglycyrol, were isolated from licorice, and they exhibited various pharmacological properties. In this report, we have accomplished the total synthesis of glycycoumarin, glycyrin, and 3-*O*-methylglycyrol in 5–7 linear steps from commercially available 2,4,6-trihydroxybenzaldehyde with yields of 12.3–21.2%. Additionally, their anti-inflammatory activities were studied and compared. Glycycoumarin, glycyrin, and 3-*O*-methylglycyrol exhibited different levels of anti-inflammatory activities, with glycyrin being the most potent. Mechanistic studies indicated that glycyrin exerted its anti-inflammatory properties by inhibiting the activation of TNF-α, IL-6, and IL-1β, making it a potential anti-inflammatory lead compound for further optimization and discovery of new agents.

## 1. Introduction

**Figure 1:**
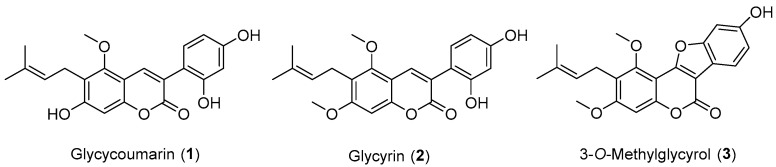
Structures of natural coumarins **1**–**3**.

Licorice (*Glycyrrhiza uralensis* Fisch), a significant traditional Chinese herbal medicine listed in the pharmacopoeia of the People’s Republic of China, has been extensively utilized in China to treat various ailments, including spleen and stomach disorders, palpitations, shortness of breath, coughs, influenza infection, and liver disease [1,2]. Licorice is also recognized for producing a variety of bioactive natural products, such as glycosides, flavonoids, and coumarins [3,4]. Glycycoumarin (Figure 1, compound **1**) is a naturally occurring coumarin that was initially extracted from licorice by Zhu et al. [5]. It has exhibited several beneficial pharmacological properties, including anti-inflammatory, antioxidant, and hepatoprotective effects [6]. Among these properties, its anti-inflammatory activity is particularly noteworthy. Glycycoumarin (**1**) has been shown to inhibit the production of nitric oxide (NO), interleukin-6 (IL-6), and prostaglandin E2 (PGE2) in LPS-induced macrophages [7]. Glycycoumarin (**1**) ameliorates alcohol-induced hepatic injury by activating Nrf2 and autophagy [8]. Furthermore, glycycoumarin (**1**) protects mice against acetaminophen-induced liver injury primarily by activating sustained autophagy [9].

As illustrated in Figure 1, the natural coumarins glycyrin (**2**) [10] and 3-*O*-methylglycyrol (**3**) were also isolated from licorice [11]. They possess chemical structures closely related to glycycoumarin (**1**). Glycyrin (**2**) has displayed therapeutic activities against metabolic syndrome [12] and exhibited antimicrobial and antiviral properties [13]. However, no data on anti-inflammatory activity for either compound **2** or **3** have been reported in the literature. Given the promising anti-inflammatory activity associated with the structurally similar natural coumarin **1**, obtaining robust and easy access to these natural products is of significant interest. However, the isolation of compounds **1**–**3** from licorice plants has been achieved with low yield. Therefore, establishing a synthetic method to obtain these compounds for further pharmacological investigation is highly desirable.

To the best of our knowledge, the first total synthesis of compound **1** was recently reported by Song et al. [14], while the syntheses of natural coumarins **2** and **3** remain undescribed. Our ongoing interest in discovering new anti-inflammatory agents from natural products prompted us to develop a reliable and efficient synthetic route for natural compounds **1**–**3** [15,16,17]. Herein, we present systematic studies toward the syntheses of natural compounds **1**–**3**, with compounds **2** and **3** being synthesized for the first time. Additionally, their anti-inflammatory activities were studied and compared.

## 2. Results and Discussion

### 2.1. Synthesis of Natural Coumarins ***1***–***3***

**Scheme 1:**
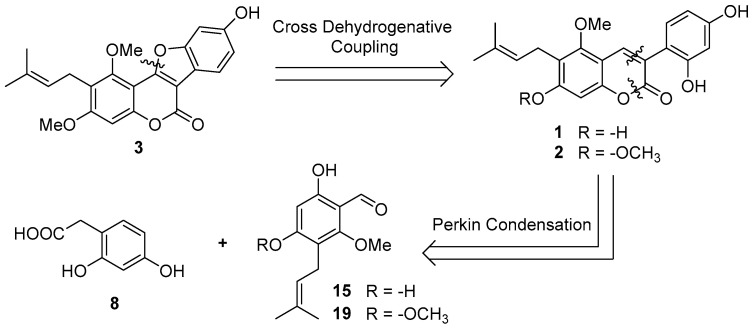
Retrosynthetic analysis of compounds **1**–**3**.

Scheme 1 outlines the retrosynthetic methodology aimed at synthesizing the desired compounds **1**–**3**. We envisaged that compound **3** could be generated through an intramolecular cross dehydrogenative C-O coupling reaction of compound **2**, and both target compounds **1** and **2** could be readily obtained through Perkin condensation between 2-(2,4-dihydroxyphenyl)acetic acid (**8**) and the corresponding 2-hydroxybenzaldehydes (**15** or **19**).

**Scheme 2:**
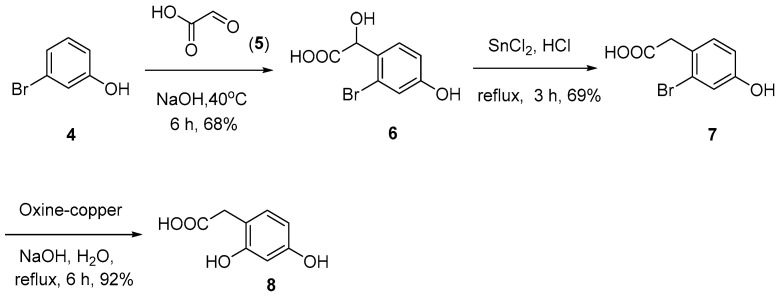
Synthesis of compound **8**.

As shown in Scheme 2, the synthesis of key intermediate **8** commenced with readily available 3-bromophenol (**4**). Treatment of compound **4** with glyoxylic acid (**5**) under basic conditions yielded compound **6** in 68%. Subsequently, the α-OH group of compound **6** was reduced using SnCl_2_/HCl to give 2-bromo-4-hydroxyphenylacetic acid (**7**), which was then hydroxylated in the presence of oxine-copper/NaOH to generate the desired intermediate, 2-(2,4-dihydroxyphenyl)acetic acid (**8**), with a 92% yield [18].

**Scheme 3:**
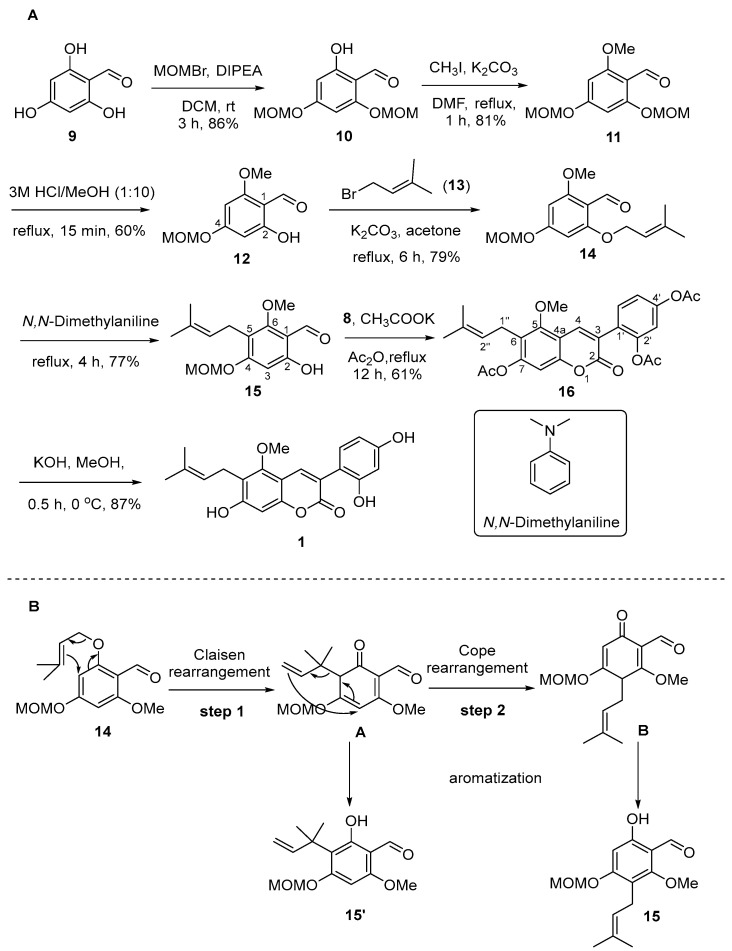
(**A**) Synthesis of glycycoumarin (**1**). (**B**) Mechanism of Claisen/Cope rearrangement.

Meanwhile, the 2- and 4-phenol groups of 2,4,6-trihydroxybenzaldehyde (9) were selectively protected, yielding compound 10 (66%) through treatment with bromo(methoxy)methane (MOMBr) in the presence of diisopropylethylamine (DIPEA) (Scheme 3A) [19]. We anticipated that the selective *O*-methylation after the coumarin scaffold formation would be challenging. Hence, the methoxyl group was introduced early, resulting in compound 11 (81%). Due to the hydrogen bond between the 2-phenol group and the formyl group (-CHO), the MOM group at the 2-phenol position can be selectively deprotected using a 3M HCl solution in MeOH (1:10), resulting in compound 12 with a 60% yield. Prenylation of compound 12 furnished prenyl ether 14 with a yield of 79% [20]. Compound 14 was then subjected to a Claisen/Cope rearrangement to give the 5-prenylbenzaldehyde 15 in *N*,*N*-dimethylaniline as a solvent [20]. Upon heating prenyl ether 14 in *N*,*N*-dimethylaniline at reflux for 1 h, we found that two products, 15 and 15′ (Scheme 3B), could be isolated [21]. The formation of the byproduct 15′ has been rationalized by assuming a dearomatized intermediate A that results from a Claisen rearrangement (step 1), and from intermediate A, the byproduct 15′ is formed through aromatization. Intermediate A may undergo a second [3,3]-sigmatropic rearrangement (step 2, Cope-rearrangement) to cyclohexadienone intermediate B, from which the desired para-product 15 is formed after aromatization. After understanding the reaction mechanism, we extended the reaction time to 5 h, successfully obtaining product 15 with a yield of 77% without the byproduct 15′.

With intermediates 2-(2,4-dihydroxyphenyl)acetic acid (**8**) and 5-prenylbenzaldehyde **15** in our hands, these two fragments were then coupled under Perkin condensation conditions, using CH_3_COOK in refluxing acetic anhydride (Ac_2_O), to give acetylated glycycoumarin (**16**) with a 61% yield [18,22,23,24]. Under these reaction conditions, the MOM protecting group at the C-7 position was concurrently transformed into the acetyl (-Ac) group. Global deacetylation with KOH in MeOH at 0 °C afforded the target compound glycycoumarin (**1**) with an 87% yield [23]. The NMR data of the synthetic sample were consistent with reported data [25] for natural glycycoumarin (**1**) (Table 1).

**Scheme 4:**
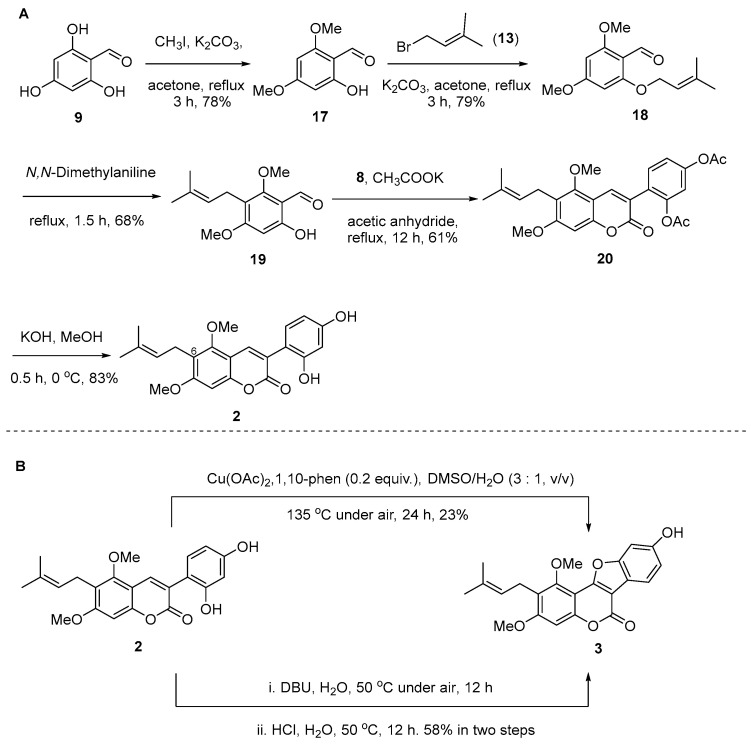
(**A**) Synthesis of glycyrin (**2**). (**B**) Synthesis of 3-*O*-methylglycyrol (**3**).

Following the successful synthesis of glycycoumarin (**1**), the syntheses of coumarins **2** and **3** were then pursued (Scheme 4). The prenylbenzaldehyde fragment of compound **2** was prepared from commercially available 2,4,6-trihydroxybenzaldehyde (**9**), which underwent a selective methylation to give 2-hydroxybenzaldehyde **17**. Compound **17** was prenylated, and the resulting prenyl ether **18** was treated with *N*,*N*-dimethylaniline at 195 °C to furnish **19** via a Claisen/Cope rearrangement. Subsequently, Perkin condensation of prenylbenzaldehyde **19** and 2-(2,4-dihydroxyphenyl)acetic acid (**8**) gave acetylated coumarin **20**, which was deacetylated to give target molecule **2** with an 83% yield. The synthetic compound **2** was characterized by ^1^H NMR and ^13^C NMR, and the data were identical with those of the natural product (Table 2) [3].

As shown in Scheme 4B, treatment of compound **2** with 0.2 equivalents of Cu(OAc)_2_ and 1,10-phen in DMSO/H_2_O (*v*/*v*, 3:1) at 135 °C generated product **3** in a low yield (23%) [22], which was difficult to isolate. We speculated that the isopentenyl group at the C-6 position was unstable under these harsh conditions, leading to the production of numerous by-products. Therefore, a 1,8-diazabicyclo [5.4.0]undec-7-ene (DBU)-promoted intramolecular dehydrogenation/oxa-Michael reaction was used to successfully obtain **3** in a moderate yield (58%) under milder conditions (DBU/H_2_O at 50 °C and then HCl at 50 °C) [18]. The NMR data of the synthetic compound **3** were consistent with the data reported for natural 3-*O*-methylglycyrol (**3**) (Table 2) [26].

### 2.2. Anti-Inflammatory Activity of Natural Coumarins ***1***–***3***

**Figure 2:**
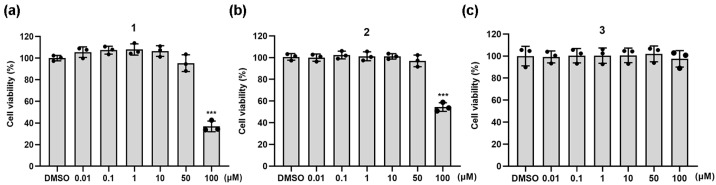
CCK-8 detected the viability of RAW264.7 cells after treatment with compounds **1** (**a**), **2** (**b**) and **3** (**c**) for 24 h. Graphic data were run in triplicate and are presented as the mean ± SD (compared with DMSO, *** *p* < 0.001).

Since nitric oxide (NO), a vital gas signaling molecule, is a unique transmitter of acute or chronic inflammation [27], the anti-inflammatory activities and cytotoxic effects of compounds **1**–**3** were evaluated by monitoring cell viability and NO inhibition in LPS-induced RAW264.7 cells, respectively. The effects of compounds **1**–**3** on cell viability were first evaluated using a CCK8 assay. RAW264.7 cells were exposed to different doses of the tested compound for 24 h. Meanwhile, 0.1% DMSO was used as the control. Significant cytotoxic effects were observed on the growth of RAW264.7 cells incubated with **1** and **2** at a dose of 100 μM (Figure 2). Cell survival was restored to that of the normal control group at reduced doses of 0.01, 0.1, 1, 10, 25, and 50 μM. The same concentration ranges were also used in cell-based studies to determine their anti-inflammatory activities.

**Figure 3:**
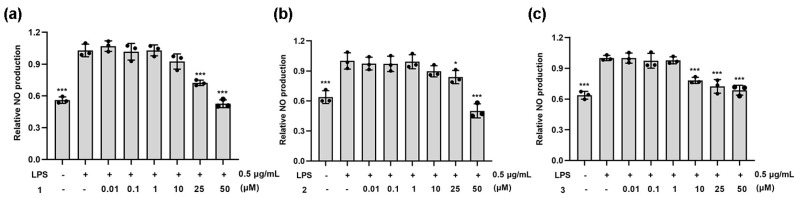
Effects of the tested compounds **1** (**a**), **2** (**b**) and **3** (**c**) on LPS-stimulated NO production in RAW264.7 cells; 0.1% DMSO was used as the control. Graphic data were run in triplicate and are presented as the mean ± SD (compared with LPS, * *p* < 0.05, *** *p* < 0.001).

To determine the anti-inflammatory activity of compounds **1**–**3**, the mouse macrophage-like cell line RAW264.7 was used. Upon treatment with LPS (0.5 μg/mL), RAW264.7 cells produced an increased amount of NO. After treatment with different concentrations of coumarins **1**–**3**, NO secretion was strongly inhibited (Figure 3). Compound **1** exhibited greater activity in inhibiting NO secretion at a dose of 25 μM and 50 μM, and the concentration of 50 μM with the inhibition rate exceeding 50%. Compound **2** showed strong inhibition of NO secretion only at 50 μM. Compound **3** inhibited NO secretion in a concentration-dependent manner.

**Figure 4:**
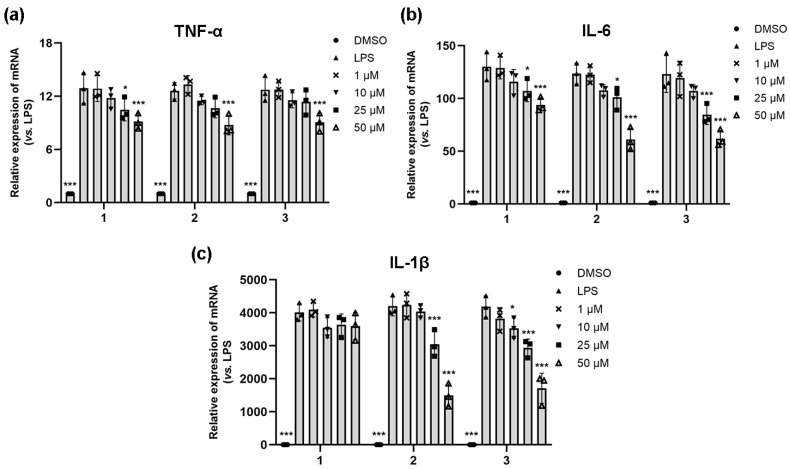
The effects of the compounds **1**–**3** on the expression of TNF-a (**a**), IL-6 (**b**), and IL-1β (**c**) detected by qRT-PCR. LPS-stimulated (0.5 μg/mL) RAW264.7 cells were treated with 1, 10, 25, and 50 μM of compounds **1**–**3** for 24 h, and 0.1% DMSO was used as the control. Graphic data were run in triplicate and are presented as the mean ± SD (compared with LPS, * *p* < 0.05, *** *p* < 0.001).

To confirm the function of compounds **1**–**3** in the LPS-induced production of pro-inflammatory cytokines, the expression levels of three critical pro-inflammatory cytokines, TNF-α, IL-6, and IL-1β mRNA, were analyzed by qRT-PCR, respectively [28]. As shown in Figure 4, LPS significantly induced the mRNA expressions of TNF-α, IL-6, and IL-1β compared with the normal control. Compound **1** demonstrated significant inhibition of IL-6 and TNF-α expression at concentrations of 25 and 50 μM but had no effect on IL-1β. Compound **2** exhibited significant inhibition of IL-6 and IL-1β expression at concentrations of 25 and 50 μM. It also significantly inhibited TNF-α at 50 μM. Compound **3** exhibited concentration-dependent inhibition of IL-6 and IL-1β, and it inhibited TNF-α only at a concentration of 50 μM.

## 3. Materials and Methods

### 3.1. General Experimental Procedures

Melting points were recorded on a Büchi B-545 melting point apparatus (Sigma-Aldrich, St. Louis, MO, USA). ^1^H NMR, ^13^C NMR spectra were recorded on a Bruker Avance 400 spectrometer (Billerica, MA, USA) or a JEOL Eclips-600 pectrometer (Akishima, Japan), and tetramethylsilane (TMS) was used as the internal reference. The HR-MS spectra were recorded by Thermo QExactive (Thermo Scientific, Waltham, MA, USA) and Agilent 6545 LC/QTOF mass spectrometers (Santa Clara, CA, USA). Column chromatography was performed on silica gel (100–200 mesh). Reagents were purchased from commercial sources and used as received, unless mentioned otherwise. The solvents were of analytical grade.

### 3.2. Synthesis and Characterization of the Compounds

#### 3.2.1. 2-(2-Bromo-4-hydroxyphenyl)-2-hydroxyacetic Acid (**6**)

3-Bromophenol (**4**, 8.65 g, 50.00 mmol, 1.0 equiv.) was added to a three-necked round-bottom flask. Once the reaction temperature reached 40 °C, a 50% aqueous solution of glyoxylic acid (**5**, 7.2 mL, 65.0 mmol, 1.3 equiv.) and an 8% aqueous NaOH (37.5 mL, 75.00 mmol, 1.5 equiv.) solution were simultaneously added slowly. The mixture was stirred for 6 h. After the reaction was complete (monitored by TLC), the mixture was cooled to room temperature and acidified to pH 1–2 using 3 M HCl (60 mL). The aqueous solution was washed with toluene (3 × 50 mL) to remove 3-bromophenol, and the product was extracted using EtOAc (3 × 50 mL). The organic layer was separated, washed with brine, dried over anhydrous Na_2_SO_4_, and concentrated in vacuo to yield compound **6** (8.40 g, 34.00 mmol, 68%) as a yellow oil. ^1^H NMR (400 MHz, DMSO-*d*_6_) *δ* 7.24 (d, *J* = 8.4 Hz, 1H), 6.97 (s, 1H), 6.77 (d, d, *J* = 8.4 Hz, 1H), 5.13 (s, 1H). The spectroscopic data correspond to reported values [22].

#### 3.2.2. 2-(2-Bromo-4-hydroxyphenyl)acetic Acid (**7**)

A solution of compound **6** (5.00 g, 20.24 mmol, 1.0 equiv.), SnCl_2_·2H_2_O (5.02 g, 22.26 mmol, 1.1 equiv.), and concentrated HCl (10.0 mL) was added to a round-bottom flask, and the mixture was stirred at 80 °C for 3 h. After the reaction was complete (monitored by TLC), H_2_O (20 mL) was added, and the mixture was heated to reflux until a clear solution was obtained. The resulting mixture was cooled to room temperature, whereupon compound **7** recrystallized to afford a white solid (3.23 g, 13.97 mmol, 69% yield). mp: 172–174 °C; ^1^H NMR (400 MHz, DMSO-*d*_6_) *δ* 7.12 (d, *J* = 8.3 Hz, 1H), 6.99 (d, *J* = 2.4 Hz, 1H), 6.71 (dd, *J* = 8.3, 2.4 Hz, 1H), 3.45 (s, 2H). The spectroscopic data correspond to reported values [22].

#### 3.2.3. 2-(2,4-Dihydroxyphenyl)acetic Acid (**8**)

Under a N_2_ atmosphere, a solution of compound **7** (2.31 g, 10.00 mmol, 1.0 equiv.), bis(8-quinolinolato)copper(II) (0.34 g, 1.00 mmol, 0.1 equiv.), NaOH (4.00 g, 100.00 mmol, 10 equiv.), and H_2_O (40 mL) was added to a round-bottom flask. The mixture was stirred at 110 °C for 6 h. After the reaction was complete (monitored by TLC), the resulting mixture was cooled to room temperature. The solid was filtered off, and the aqueous solution was acidified to pH 1-2 using 3 M HCl (40 mL). The product was extracted using EtOAc (3 × 50 mL). The organic layer was separated, washed with brine, dried over anhydrous Na_2_SO_4_, and concentrated in vacuo to yield compound **8** (1.55 g, 9.20 mmol, 92%) as a yellow oil. ^1^H NMR (400 MHz, DMSO-*d*_6_) *δ* 9.03 (s, 1H), 6.79 (d, *J* = 8.1 Hz, 1H), 6.24 (d, *J* = 2.4 Hz, 1H), 6.12 (dd, *J* = 8.1, 2.4 Hz, 1H), 3.29 (s, 2H). The spectroscopic data correspond to reported values [22].

#### 3.2.4. 2-Hydroxy-4,6-bis(methoxymethoxy)benzaldehyde (**10**)

Under a N_2_ atmosphere, a solution of 2,4,6-trihydroxybenzaldehyde (**9**, 5.00 g, 32.44 mmol, 1.0 equiv.) and DIPEA (22.60 mL, 129.76 mmol, 4.0 equiv.) in CH_2_Cl_2_ (200 mL) was cooled to 0 °C, and MOMBr (5.30 mL, 64.88 mmol, 2.0 equiv.) was added. The resulting mixture was stirred at room temperature for 3 h. After the reaction was complete (monitored by TLC), H_2_O (100 mL) was added, and the mixture was extracted with CH_2_Cl_2_ (3 × 50 mL). The organic layer and extracts were combined, dried, and evaporated to give a red oil, which was chromatographed on silica gel (petroleum ether/EtOAc = 50/1) to yield compound **10** (6.76 g, 27.90 mmol, 86%) as a white solid. mp: 67–68 °C. ^1^H NMR (400 MHz, CDCl_3_) *δ* 12.31 (s, 1H), 10.19 (s, 1H), 6.28 (d, *J* = 2.1 Hz, 1H), 6.25 (d, *J* = 2.1 Hz, 1H), 5.26 (s, 2H), 5.20 (s, 2H), 3.53 (s, 3H), 3.50 (s, 3H). The spectroscopic data correspond to reported values [29].

#### 3.2.5. 2-Methoxy-4,6-bis(methoxymethoxy)benzaldehyde (**11**)

Under a N_2_ atmosphere, a solution of compound **10** (3.50 g, 14.45 mmol, 1.0 equiv.), K_2_CO_3_ (9.99 g, 72.25 mmol, 5.0 equiv.), and CH_3_I (2.25 mL, 36.13 mmol, 2.5 equiv.) in anhydrous DMF (50 mL) was stirred at 153 °C for 1 h. After the reaction was complete (monitored by TLC), the mixture was cooled to room temperature, quenched with water (20 mL), and extracted with EtOAc (3 × 50 mL). The combined organic layers were washed with brine, dried over Na_2_SO_4_, and concentrated in vacuo. The crude material was purified by flash chromatography (PE/EtOAc = 20/1) to yield compound **11** (3.00 g, 11.70 mmol, 81% yield) as yellow oil. ^1^H NMR (400 MHz, CDCl_3_) *δ* 10.40 (s, 1H), 6.46 (d, *J* = 1.8 Hz, 1H), 6.31 (d, *J* = 1.8 Hz, 1H), 5.25 (s, 2H), 5.22 (s, 2H), 3.89 (s, 3H), 3.51 (s, 3H), 3.50 (s, 3H); ^13^C NMR (100 MHz, CDCl_3_) *δ* 187.8, 163.7, 163.5, 161.7, 110.2, 95.4, 94.9, 94.2, 93.3, 56.6, 56.5, 56.1. HRMS (ESI) calculated for C_12_H_17_O_6_^+^ [M + H]^+^ 257.1020, found 257.1014.

#### 3.2.6. 2-Hydroxy-6-methoxy-4-(methoxymethoxy)benzaldehyde (**12**)

A solution of compound **11** (3.00 g, 11.71 mmol) and 3 M HCl (27 mL) in anhydrous MeOH (270 mL) was stirred at 66 °C for 15 min. After the reaction was complete (monitored by TLC), the mixture was poured into ice-cold water, and the resulting solution was adjusted to pH 7.0 with saturated NaHCO_3_ (100 mL) and extracted three times with EtOAc (3 × 50 mL). The combined organic layers were washed with brine, dried over Na_2_SO_4_, and concentrated in vacuo. The crude material was purified by flash chromatography (PE/EtOAc = 50/1) to yield compound **12** (1.49 g, 7.03 mmol, 60%) as a white solid. mp: 63–65 °C; ^1^H NMR (400 MHz, CDCl3) *δ* 12.38 (s, 1H), 10.14 (s, 1H), 6.19 (d, *J* = 1.9 Hz, 1H), 6.04 (d, *J* = 1.9 Hz, 1H), 5.21 (s, 2H), 3.88 (s, 3H), 3.50 (s, 3H); ^13^C NMR (100 MHz, CDCl_3_) *δ* 192.1, 165.9, 165.7, 163.8, 106.6, 95.7, 94.1, 91.2, 56.5, 55.8. HRMS (ESI) calculated for C_10_H_13_O_5_^+^ [M + H]^+^ 213.0758, found 213.0752.

#### 3.2.7. 2-Methoxy-4-(methoxymethoxy)-6-((3-methylbut-2-en-1-yl)oxy)benzaldehyde (**14**)

Under a N_2_ atmosphere, to a solution of **12** (1.70 g, 8.01 mmol, 1.0 equiv.) and K_2_CO_3_ (2.21 g, 16.02 mmol, 2.0 equiv.) in acetone (20 mL) was added **13** (1.41 mL, 12.02 mmol, 1.5 equiv.) and refluxed for 6 h. After the reaction was completed (detected by TLC). The mixture was cooled to room temperature, filtered, and concentrated in vacuo. The crude material was purified by flash chromatography (PE/EtOAc = 20/1) to give **14** (1.77 g, 6.33 mmol, 79%) as yellow oil. ^1^H NMR (400 MHz, CDCl_3_) *δ* 10.39 (s, 1H), 6.26 (d, *J* = 1.9 Hz, 1H), 6.23 (d, *J* = 1.9 Hz, 1H), 5.48 (t, *J* = 6.6 Hz, 1H), 5.22 (s, 2H), 4.60 (d, *J* = 6.6 Hz, 2H), 3.88 (s, 3H), 3.51 (s, 3H), 1.80 (s, 3H), 1.75 (s, 3H); ^13^C NMR (100 MHz, CDCl_3_) *δ* 188.0, 163.8, 163.7, 163.4, 138.4, 119.0, 109.8, 94.2, 93.3, 92.2, 66.0, 56.4, 56.0, 25.8, 18.3.HRMS (ESI) calculated for C_15_H_21_O_5_^+^ [M + H]^+^ 281.1384, found 281.1378.

#### 3.2.8. 6-Hydroxy-2-methoxy-4-(methoxymethoxy)-3-(3-methylbut-2-en-1-yl)benzaldehyde (**15**)

Under a N_2_ atmosphere, a solution of compound **14** (1.00 g, 3.57 mmol) in *N*,*N*-dimethylaniline (5 mL) was refluxed for 4 h. After the reaction was complete (monitored by TLC), the mixture was cooled to room temperature, and the resulting solution was adjusted to pH 7.0 with 3 M HCl (5 mL) and extracted three times with EtOAc (3 × 50 mL). The combined organic layers were washed with brine, dried over Na_2_SO_4_, and concentrated in vacuo. The crude material was purified by flash chromatography (PE/EtOAc = 50/1) to give **15** (0.77 g, 2.75 mmol, 77%) as yellow oil. ^1^H NMR (400 MHz, CDCl_3_) *δ* 11.99 (s, 1H), 10.07 (s, 1H), 6.45 (s, 1H), 5.25 (s, 2H), 5.20–5.11 (m, 1H), 3.87 (s, 3H), 3.49 (s, 3H), 3.29 (d, *J* = 6.9 Hz, 2H), 1.79 (s, 3H), 1.70 (s, 3H); ^13^C NMR (100 MHz, CDCl_3_) *δ* 192.9, 163.7, 163.6, 162.0, 131.8, 122.6, 115.9, 109.5, 98.13, 94.0, 64.4, 56.4, 25.7, 22.3, 17.8.HRMS (ESI) calculated for C_15_H_21_O_5_^+^ [M + H]^+^ 281.1384, found 281.1377.

#### 3.2.9. 4-(7-Acetoxy-5-methoxy-6-(3-methylbut-2-en-1-yl)-2-oxo-2H-chromen-3-yl)-1,3-phenylene Diacetate (**16**)

Under a N_2_ atmosphere, a solution of compound **15** (0.28 g, 1.00 mmol, 1.0 equiv.), compound **8** (0.84 g, 5.00 mmol, 5.0 equiv.), and anhydrous CH_3_COOK (0.18 g, 1.80 mmol, 1.8 equiv.) in Ac_2_O (1.2 mL) was refluxed for 12 h. After the reaction was complete (monitored by TLC), the mixture was poured into ice-cold water, and the resulting solution was adjusted to pH 7.0 with saturated NaHCO_3_ (5 mL) and extracted three times with EtOAc (3 × 50 mL). The combined organic layers were washed with brine, dried over Na_2_SO_4_, and concentrated in vacuo. The crude material was purified by flash chromatography (PE/EtOAc = 10/1) to yield compound **16** (0.3 g, 0.61 mmol, 61%) as a white solid. mp: 71–73 °C; ^1^H NMR (400 MHz, CDCl_3_) *δ* 7.94 (s, 1H), 7.47 (d, *J* = 9.1 Hz, 1H), 7.14-7.09 (m, 2H), 6.95 (s, 1H), 5.08 (t, *J* = 6.6 Hz, 1H), 3.87 (s, 3H), 3.35 (d, *J* = 6.6 Hz, 2H), 2.35 (s, 3H), 2.34 (s, 3H), 2.21 (s, 3H), 1.79 (s, 3H), 1.72 (s, 3H); ^13^C NMR (100 MHz, CDCl_3_) *δ* 169.0, 168.8, 168.5, 159.3, 156.0, 152.8, 152.5, 151.3, 149.0, 137.4, 132.9, 131.4, 125.5, 124.4, 123.9, 121.4, 119.3, 117.0, 112.1, 107.5, 63.5, 25.6, 23.5, 21.1, 21.0, 20.9, 18.0. HRMS (ESI) calculated for C_27_H_27_O_9_^+^ [M + H]^+^ 495.1650, found 495.1642.

#### 3.2.10. Glycycoumarin (**1**)

A solution of compound **16** (0.20 g, 0.40 mmol, 1.0 equiv.) in MeOH (5 mL) was treated with KOH (0.07 g, 1.20 mmol, 3 equiv.). The mixture was stirred at 0 °C for 0.5 h. After the reaction was complete (monitored by TLC), the mixture was poured into ice-cold water. The resulting solution was adjusted to pH 7.0 with 3 M HCl (5 mL) and extracted with EtOAc (3 × 20 mL). The organic layer was combined, washed with saturated aqueous NaCl solution, and dried with anhydrous Na_2_SO_4_. The solvents were then removed in vacuo. The crude material was purified by flash chromatography (PE/EtOAc = 5/1) to yield compound **1** (0.13 g, 0.35 mmol, 87%) as a yellow solid. mp: 234–236 °C; ^1^H NMR (400 MHz, DMSO-*d*_6_) *δ* 10.59 (s, 1H), 9.40 (s, 2H), 7.82 (s, 1H,), 7.11 (d, *J* = 8.3 Hz, 1H), 6.61 (s, 1H), 6.37 (d, *J* = 2.3 Hz, 1H), 6.27 (dd, *J* = 8.3, 2.3 Hz, 1H), 5.16 (t, *J* = 6.9 Hz), 3.77 (s, 3H), 3.27 (d, *J* = 6.9 Hz, 2H), 1.74 (s, 3H), 1.64 (s, 3H); ^13^C NMR (100 MHz, DMSO-*d*_6_) *δ* 160.0, 159.2, 158.3, 156.0, 155.2, 152.9, 136.4, 131.5, 130.7, 122.6, 120.3, 118.3, 113.4, 106.2, 106.1, 102.6, 97.9, 62.8, 25.4, 22.3, 17.7.

#### 3.2.11. 2-Hydroxy-4,6-dimethoxybenzaldehyde (**17**)

Under a N_2_ atmosphere, a suspension of 2,4,6-trihydroxybenzaldehyde (**9**, 5.00 g, 32.44 mmol, 1.0 equiv.), CH_3_I (4.45 mL, 71.40 mmol, 2.2 equiv.), and K_2_CO_3_ (8.97 g, 64.89 mmol, 2.0 equiv.) in acetone (50 mL) was stirred at 50 °C for 3 h. After the reaction was complete (monitored by TLC), the mixture was cooled to room temperature, quenched with water (50 mL), and extracted with EtOAc (3 × 50 mL). The combined organic layers were washed with brine, dried over Na_2_SO_4_, and concentrated in vacuo. The crude material was purified by flash chromatography (PE/EtOAc = 20/1) to yield compound **17** (4.61 g, 25.30 mmol, 78%) as a yellow solid. mp: 67–69 °C; ^1^H NMR (400 MHz, CDCl_3_) *δ* 12.53 (s, 1H), 10.10 (s, 1H), 6.02 (d, *J* = 2.1 Hz, 1H), 5.92 (d, *J* = 2.1 Hz, 1H), 3.86 (s, 3H), 3.85 (s, 3H). The spectroscopic data correspond to reported values [30].

#### 3.2.12. 2,4-Dimethoxy-6-((3-methylbut-2-en-1-yl)oxy)benzaldehyde (**18**)

Under a N_2_ atmosphere, a solution of compound **17** (1.83 g, 10.00 mmol) and K_2_CO_3_ (2.76 g, 20.00 mmol, 2.0 equiv.) in acetone (20 mL) was treated with compound **13** (1.76 mL, 15.00 mmol, 1.5 equiv.) and refluxed for 3 h. After the reaction was complete (monitored by TLC), the mixture was cooled to room temperature, filtered, and concentrated in vacuo. The crude material was purified by flash chromatography (PE/EtOAc = 10/1) to yield compound **18** (1.98 g, 7.90 mmol, 79%) as white solid. mp: 62–63 °C; ^1^H NMR (400 MHz, CDCl_3_) *δ* 10.37 (s, 1H), 6.09 (s, 1H), 6.07 (s, 1H), 5.48 (t, *J* = 6.1 Hz), 4.60 (d, *J* = 6.1 Hz), 3.88 (s, 3H), 3.87 (s, 3H), 1.79 (s, 3H), 1.75 (s, 3H). The spectroscopic data correspond to reported values [31].

#### 3.2.13. 6-Hydroxy-2,4-dimethoxy-3-(3-methylbut-2-en-1-yl)benzaldehyde (**19**)

Under a N_2_ atmosphere, a solution of compound **18** (1.00 g, 4.00 mmol) in *N*,*N*-dimethylaniline (5 mL) was refluxed for 1.5 h. After the reaction was complete (monitored by TLC), the mixture was cooled to room temperature, and the resulting solution was adjusted to pH 7.0 with 3 M HCl (5 mL) and extracted three times with EtOAc (3 × 50 mL). The combined organic layers were washed with brine, dried over Na_2_SO_4_, and concentrated in vacuo. The crude material was purified by flash chromatography (PE/EtOAc = 50/1) to yield compound **19** (0.68 g, 2.72 mmol, 68%) as yellow oil. ^1^H NMR (400 MHz, CDCl_3_) *δ* 12.14 (s, 1H), 10.04 (s, 1H), 6.24 (s, 1H), 5.14 (t, *J* = 7.0 Hz, 1H), 3.88 (s, 3H), 3.86 (s, 3H), 3.26 (d, *J* = 7.0 Hz, 2H), 1.78 (s, 3H), 1.70 (s, 3H); ^13^C NMR (100 MHz, CDCl_3_) *δ* 192.7, 166.4, 164.1, 161.7, 131.8, 122.5, 115.5, 108.8, 95.5, 64.3, 56.0, 25.7, 22.1, 17.8. HRMS (ESI) calculated for C_14_H_19_O_4_^+^ [M + H]^+^ 251.1278, found 251.1272.

#### 3.2.14. 4-(5,7-Dimethoxy-6-(3-methylbut-2-en-1-yl)-2-oxo-2H-chromen-3-yl)-1,3-phenylene Diacetate (**20**)

Under a N_2_ atmosphere, a solution of **19** (0.25 g, 1.00 mmol, 1.0 equiv.), **8** (0.84 g, 5.00 mmol, 5 equiv.), and anhydrous CH_3_COOK (0.18 g, 1.80 mmol, 1.8 equiv.) in Ac_2_O (1.2 mL) was refluxed for 12 h. After the reaction was completed (detected by TLC), the mixture was poured into ice-cold water and the obtained solution was adjusted to pH = 7.0 with saturated NaHCO_3_ (5 mL) and extracted three times with EtOAc (3 × 50 mL). The combined organic layers were washed with brine, dried over Na_2_SO_4_, and concentrated in vacuo. The crude material was purified by flash chromatography (PE/EtOAc = 10/1) to give **20** (0.28 g, 0.61 mmol, 61%) as yellow solid. mp: 160–161 °C; ^1^H NMR (400 MHz, CDCl_3_) *δ* 7.91 (s, 1H), 7.48 (dd, *J* = 8.0, 0.8 Hz, 1H), 7.13-7.07 (m, 2H), 6.69 (s, 1H), 5.16 (t, *J* = 6.9 Hz, 1H), 3.92 (s, 3H), 3.85 (s, 3H), 3.39 (d, *J* = 6.9 Hz, 2H), 2.34 (s, 3H), 2.20 (s, 3H), 1.80 (s, 3H), 1.71 (s, 3H); ^13^C NMR (100 MHz, CDCl_3_) *δ* 169.0, 168.6, 161.9, 160.0, 155.4, 154.5, 151.0, 148.9, 138.3, 132.2, 131.5, 126.0, 122.1, 120.6, 120.4, 119.2, 116.9, 107.4, 95.1, 63.2, 56.2, 25.8, 22.8, 21.2, 21.0, 17.9. HRMS (ESI) calculated for C_26_H_27_O_8_^+^ [M + H]^+^ 467.1701, found 467.1691. The spectroscopic data correspond to reported values [10].

#### 3.2.15. Glycyrin (**2**)

A solution of compound **20** (0.12 g, 0.26 mmol, 1.0 equiv.) in MeOH (5 mL) was treated with KOH (0.03 g, 0.52 mmol, 2 equiv.). The mixture was stirred at 0 °C for 0.5 h. After the reaction was complete (monitored by TLC), the mixture was poured into ice-cold water. The resulting solution was adjusted to pH 7.0 with 3 M HCl (2 mL) and extracted with EtOAc (3 × 20 mL). The organic layers were combined, washed with saturated aqueous NaCl solution, and dried with anhydrous Na_2_SO_4_. The solvents were then removed in vacuo. The crude material was purified by flash chromatography (PE/EtOAc = 5/1) to yield compound **2** (0.083 g, 0.217 mmol, 83%) as a yellow solid. mp: 203–205 °C; ^1^H NMR (400 MHz, DMSO-*d*_6_) *δ* 9.46 (s, 1H), 9.44 (s, 1H), 7.86 (s, 1H), 7.14 (d, *J* = 8.4 Hz, 1H), 6.89 (s, 1H), 6.38 (d, *J* = 2.3 Hz, 1H), 6.28 (dd, *J* = 8.4, 2.3 Hz, 1H), 5.11 (t, *J* = 7.0 Hz, 1H), 3.89 (s, 3H), 3.78 (s, 3H), 3.29 (d, *J* = 7.0 Hz, 2H), 1.74 (s, 3H), 1.64 (s, 3H); ^13^C NMR (100 MHz, DMSO-*d*_6_) *δ* 160.9, 160.4, 159.0, 156.6, 155.0, 153.8, 136.6, 132.1, 131.6, 122.8, 121.9, 119.6, 113.7, 107.5, 106.7, 103.2, 95.7, 63.4, 56.9, 25.9, 22.7, 18.2.

#### 3.2.16. 3-*O*-Methylglycyrol (**3**)

A solution of compound **2** (0.10 g, 0.26 mmol, 1.0 equiv.) in H_2_O (2 mL) was treated with DBU (0.18 mL, 1.30 mmol, 5 equiv.). The mixture was stirred at 50 °C in a water bath for 12 h. After the reaction was complete (monitored by TLC), the mixture was acidified to pH 1.0 with 3 M HCl (5 mL) and stirred for an additional 12 h. The mixture was then extracted with EtOAc (3 × 15 mL). The organic layers were combined, washed with saturated aqueous NaCl solution, and dried with anhydrous Na_2_SO_4_. The solvents were then removed in vacuo. The crude material was purified by flash chromatography (PE/EtOAc = 5/1) to yield compound **3** (0.06 g, 0.15 mmol, 58%). mp: 255–257 °C; ^1^H NMR (400 MHz, DMSO-*d*_6_) *δ* 10.06 (s, 1H), 7.73 (d, *J* = 8.4 Hz, 1H), 7.18 (d, *J* = 2.0 Hz, 1H), 7.04 (s, 1H), 6.97 (dd, *J* = 8.4, 2.0 Hz, 1H), 5.14 (t, *J* = 7.2 Hz, 1H), 3.91 (s, 6H), 3.33 (d, *J* = 7.2 Hz, 2H), 1.76 (s, 3H), 1.64 (s, 3H); ^13^C NMR (100 MHz, DMSO-*d*_6_) *δ* 161.0, 158.3, 157.9, 157.6, 156.7, 153.8, 153.6, 131.8, 122.6, 121.1, 121.0, 114.6 (2 × C), 103.4, 101.3, 99.0, 96.9, 63.0, 57.0, 25.9, 22.4, 18.2.

#### 3.2.17. Cell Culture

The RAW264.7 cell line was obtained from Procell (Wuhan, China), authenticated by short tandem repeat profiling (STR), and examined for mycoplasma contamination. RAW264.7 cells were cultured in Dulbecco’s Modified Eagle’s Medium (DMEM; Hyclone, UT, USA), which was supplemented with 10% fetal bovine serum (FBS; Anweisci, Shanghai, China), and maintained at 37 °C and 5% CO_2_.

#### 3.2.18. Cell Viability Assay

RAW264.7 cells were seeded into 96-well plates (1 × 10^3^ cells/well) overnight and treated with 0.1% DMSO (negative control) and different concentration of test compounds. After 24 h, 10% (*v*/*v*) CCK-8 (Beyotime, Shanghai, China) was added to each well and incubated for 1 h. Fluorescence intensities were measured by using a Varioskan Flash Multimode Reader (Thermo Fisher Scientific, Waltham, MA, USA) at 450 nm.

#### 3.2.19. NO Determination

RAW264.7 cells were seeded in 96-well plate at a density of 1 × 10^5^ cells/well. The cells were treated with different concentrations of the test compounds; after 1 h, the cells were stimulated with LPS (0.5 μg/mL) and then incubated for 24 h. The nitrite accumulated in the culture medium was measured as an indicator of NO production based on a diazotisation reaction using the Griess reagent system as previously described [32].

#### 3.2.20. Real-Time Quantitative PCR

TRIzol reagent (Invitrogen, Carlsbad, CA, USA) was using to extract total cellular RNA according to the manufacturer’s instructions. RNA concentration was determined by examining the absorbance at 260 nm using a Varioskan Flash Multimode Reader (Thermo Fisher Scientific). Total RNA (2 μg) was reverse-transcribed using TransScript^®^ All-in-One First-Strand cDNA Synthesis SuperMix for qPCR (Cat No: AT341-01, TransGen Biotech, Beijing, China). Equal amounts of cDNA were subjected to RT-qPCR with TransStart^®^ Top Green qPCR SuperMix (Cat No: AQ131-02, TransGen Biotech, Beijing, China) using a Chromo4 detection system (Bio-Rad, California, USA). GAPDH served as reference genes to eliminate differences in the number of cells. Quantitative analyses were performed using the threshold cycle number (Ct), where the signal was detected above the background and was in the exponential phase. Relative RNA expression was analyzed by 2^−△△C(t)^, and DMSO was used as a control. The sequences of primers used are showed in Table 3.

## 4. Conclusions

In summary, the total synthesis of glycycoumarin (**1**), glycyrin (**2**), and 3-*O*-methylglycyrol (**3**) has been accomplished in 5–7 steps from commercially available 2,4,6-trihydroxybenzaldehyde (**9**) with yields of 13.5%, 21.2%, and 12.3%, respectively. Notably, glycyrin (**2**) and 3-*O*-methylglycyrol (**3**) were synthesized for the first time. Our synthetic strategy features a Perkin condensation to establish the 3-phenyl-2H-chromen-2-one framework and a Claisen/Cope rearrangement to introduce the isopentene group to the coumarin core. Furthermore, the anti-inflammatory potencies of the synthetic natural products **1**–**3** were investigated using various in vitro systems, including the inhibition of NO production in LPS-induced RAW264.7 cells and the inhibition of three critical pro-inflammatory cytokines (TNF-α, IL-6, and IL-1β). Among compounds **1**–**3**, the anti-inflammatory activities of glycyrin (**2**) and 3-*O*-methylglycyrol (**3**) were first reported. Generally, compounds **1**–**3** exhibited different levels of anti-inflammatory activities, with compound **2** being the most potent. Mechanistic studies indicated that compound **2** exerted its anti-inflammatory property by inhibiting the activation of TNF-α, IL-6, and IL-1β. Hence, compound **2** could be a potential anti-inflammatory lead compound for further optimization and discovery of new agents.

## Figures and Tables

**Table 1:** Comparison of the ^13^C NMR Data of synthesized compound **1** with literature data [25].

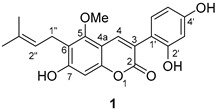			
No.	Natural Product (*δ*_1_) ^1^	Synthetic Sample (*δ*_1_) ^1^	∆ = *δ*_1_ − *δ*_2_
2	159.9	160.0	−0.1
3	120.3	120.3	0
4	136.3	136.4	−0.1
4a	106.1	106.2	−0.1
5	158.3	158.3	0
6	113.4	113.4	0
7	159.2	159.2	0
8	97.8	97.9	−0.1
8a	152.9	152.9	0
1′	118.3	118.3	0
2′	155.9	156.0	−0.1
3′	102.6	102.6	0
4′	155.2	155.2	0
5′	105.8	106.1	−0.3
6′	131.5	131.5	0
1″	22.2	22.3	−0.1
2″	122.6	122.6	0
3″	130.6	130.7	−0.1
4″	17.7	17.7	0
5″	25.4	25.4	0
-OCH_3_	62.7	62.8	−0.1

^1^ The NMR data were recorded in DMSO-*d*_6._

**Table 2:** Comparison of the ^13^C NMR Data of synthesized compounds **2** and **3** with literature data [3,26].

	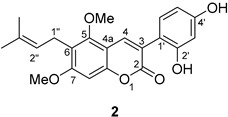			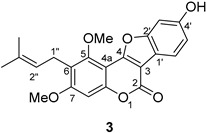		
No.	Natural Product (*δ*_1_) ^1^	Synthetic Sample (*δ*_1_) ^1^	∆ = *δ*_1_ − *δ*_2_	Natural Product (*δ*_1_) ^1^	Synthetic Sample (*δ*_1_) ^1^	∆ = *δ*_1_ − *δ*_2_
2	160.9	160.9	0	157.9	157.9	0
3	121.8	121.9	−0.1	103.4	103.4	0
4	136.4	136.6	−0.2	158.3	158.3	0
4a	107.5	107.5	0	101.3	101.3	0
5	155.0	155.0	0	153.6	153.6	0
6	119.6	119.6	0	121.0	121.0	0
7	158.9	159.0	−0.1	161.0	161.0	0
8	95.6	95.7	−0.1	97.0	96.9	−0.1
8a	153.7	153.8	−0.1	153.8	153.8	0
1′	113.7	113.7	0	114.6	114.6	0
2′	156.5	156.6	−0.1	156.7	156.7	0
3′	103.1	103.2	−0.1	99.0	99.0	0
4′	160.3	160.4	−0.1	157.6	157.6	0
5′	106.7	106.7	0	114.6	114.6	0
6′	132.0	132.1	−0.1	121.1	121.1	0
1″	22.7	22.7	0	22.5	22.4	0.1
2″	122.7	122.8	−0.1	122.6	122.6	0
3″	131.5	131.6	−0.1	131.8	131.8	0
4″	18.1	18.2	−0.1	18.1	18.2	−0.1
5″	25.8	25.9	−0.1	25.9	25.9	0
-OCH_3_	63.4	63.4	0	57.0	57.0	0
-OCH_3_	56.8	56.9	−0.1	63.0	63.0	0

^1^ The NMR data were recorded in DMSO-*d*_6._

**Table 3:** Primers used in quantitative RT-PCR.

Name	Sequence (5′-3′)
TNF	Forward 5′- GCCTCTTCTCATTCCTGCTTGTGG -3′
	Reverse 5′- GTGGTTTGTGAGTGTGAGGGTCTG -3′
IL-6	Forward 5′- CTTCTTGGGACTGATGCTGGTGAC -3′
	Reverse 5′- AGGTCTGTTGGGAGTGGTATCCTC -3′
IL-1β	Forward 5′- TCGCAGCAGCACATCAACAAGAG -3′
	Reverse 5′- AGGTCCACGGGAAAGACACAGG -3′
GAPDH	Forward 5′- GGCAAATTCAACGGCACAGTCAAG -3′
	Reverse 5′- TCGCTCCTGGAAGATGGTGATGG -3′

## Data Availability

Data are contained within the article and Supplementary Materials.

## Supplementary Materials

The following supporting information can be downloaded at: https://www.mdpi.com/article/10.3390/molecules29163942/s1. ^1^H NMR and ^13^C NMR spectra for the synthesized compounds.
