# Supplementary material for: Total Syntheses and Anti-Inflammatory Studies of Three Natural Coumarins: Glycycoumarin, Glycyrin, and 3-O-Methylglycyrol
Source: Molecules. 2024 Aug 21;29(16):3942. doi: 10.3390/molecules29163942 (PMC11357431; doi:10.3390/molecules29163942)
Supplement: Supplementary file 1 [file molecules-29-03942-s001.zip › molecules-3155157-supplementary.pdf]

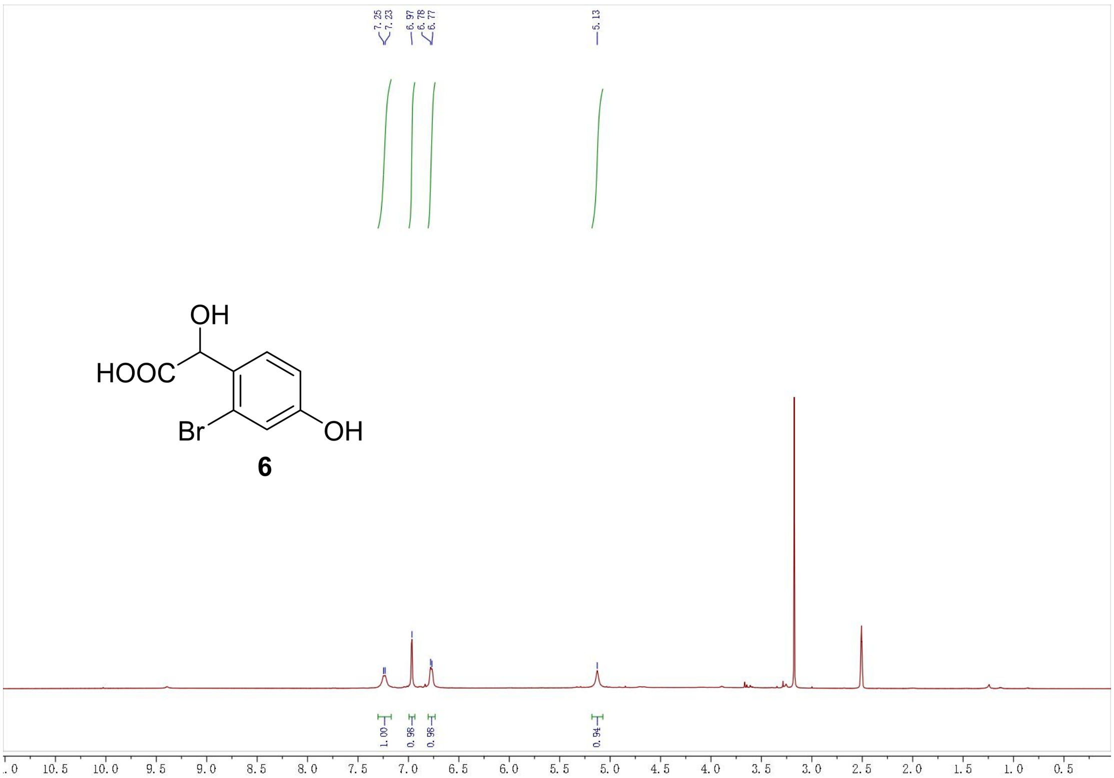

Figure S1 ^{1}H NMR (400 MHz, DMSO-d_6) of compound **6**

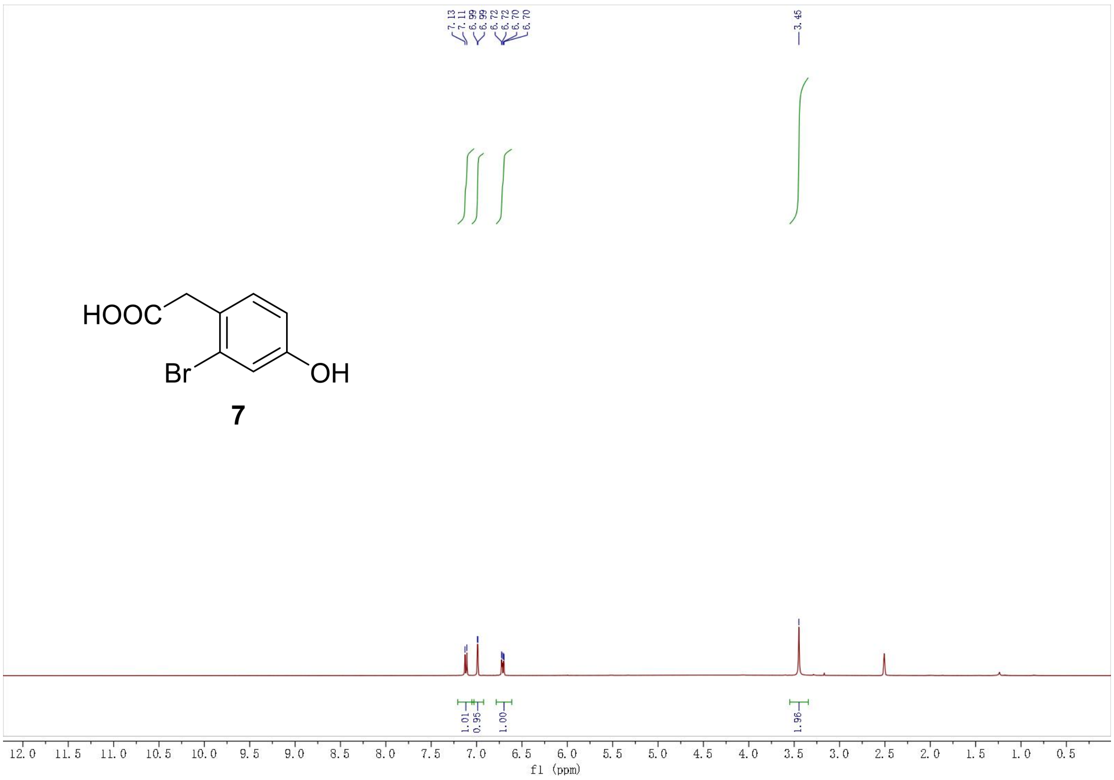

Figure S2 ^{1}H NMR (400 MHz, DMSO-d_6) of compound **7**

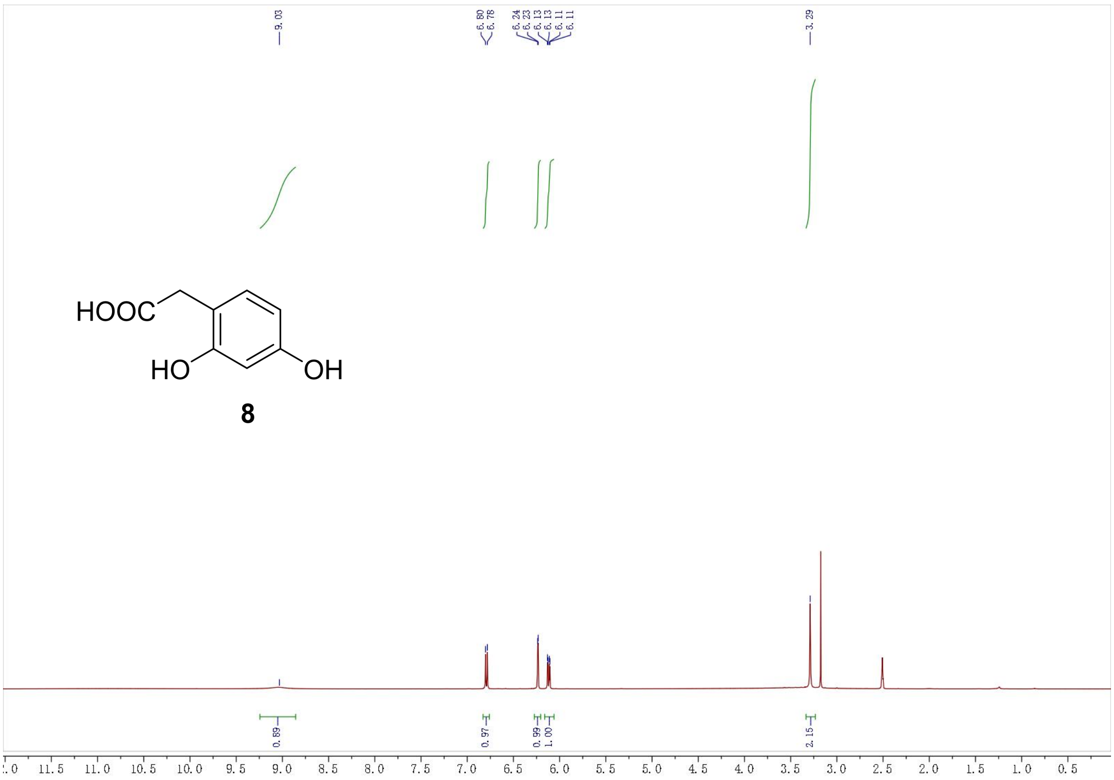

Figure S3 ^{1}H NMR (400 MHz, DMSO-d_6) of compound **8**

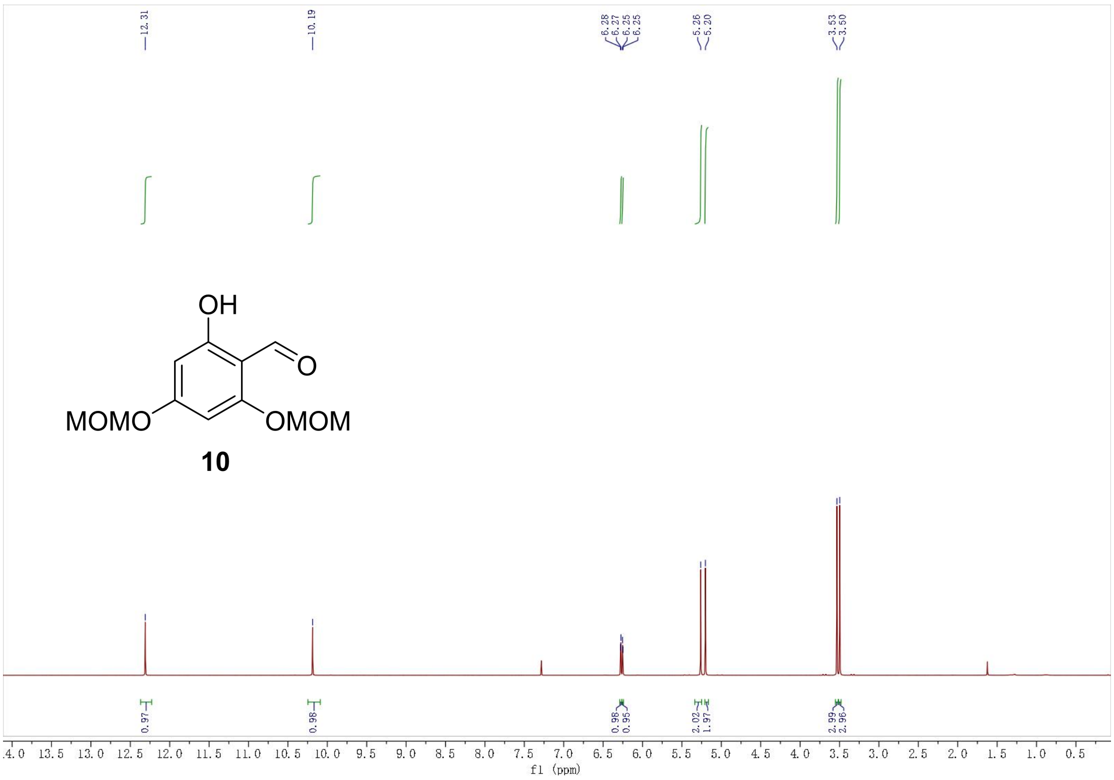

Figure S4 ^{1}H NMR (400 MHz, $CDCl_3$) of compound **10**

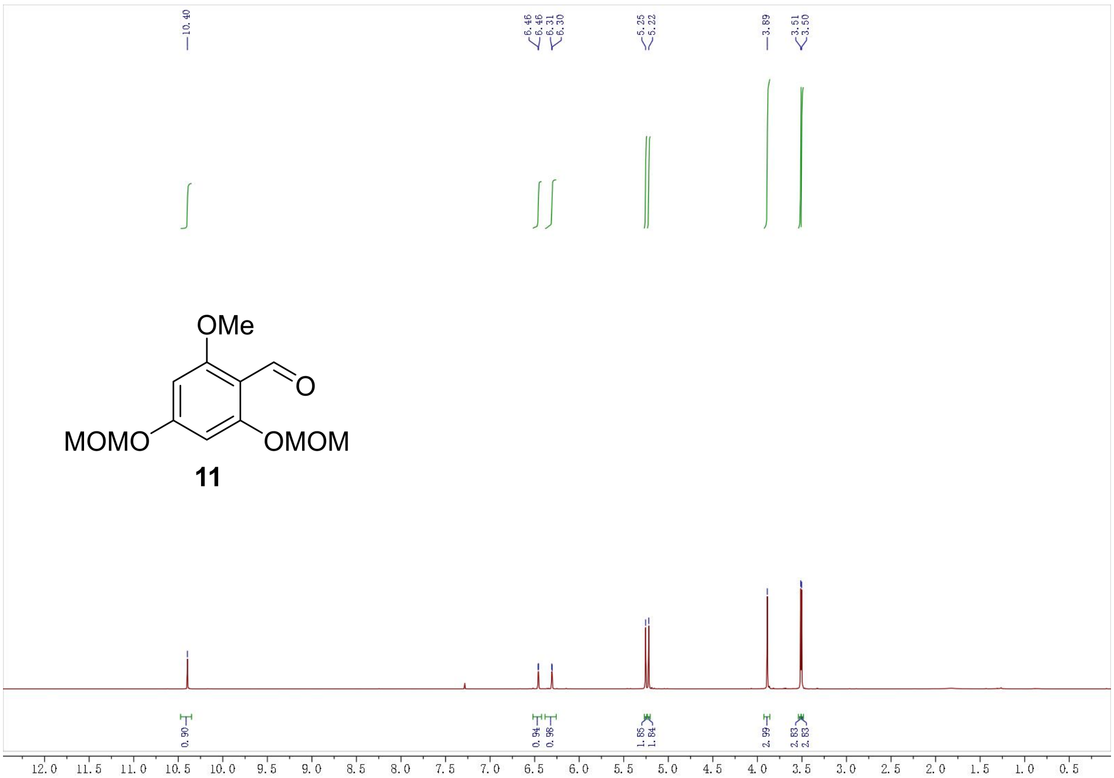

Figure S5 ^{1}H NMR (400 MHz, $CDCl_3$) of compound **11**

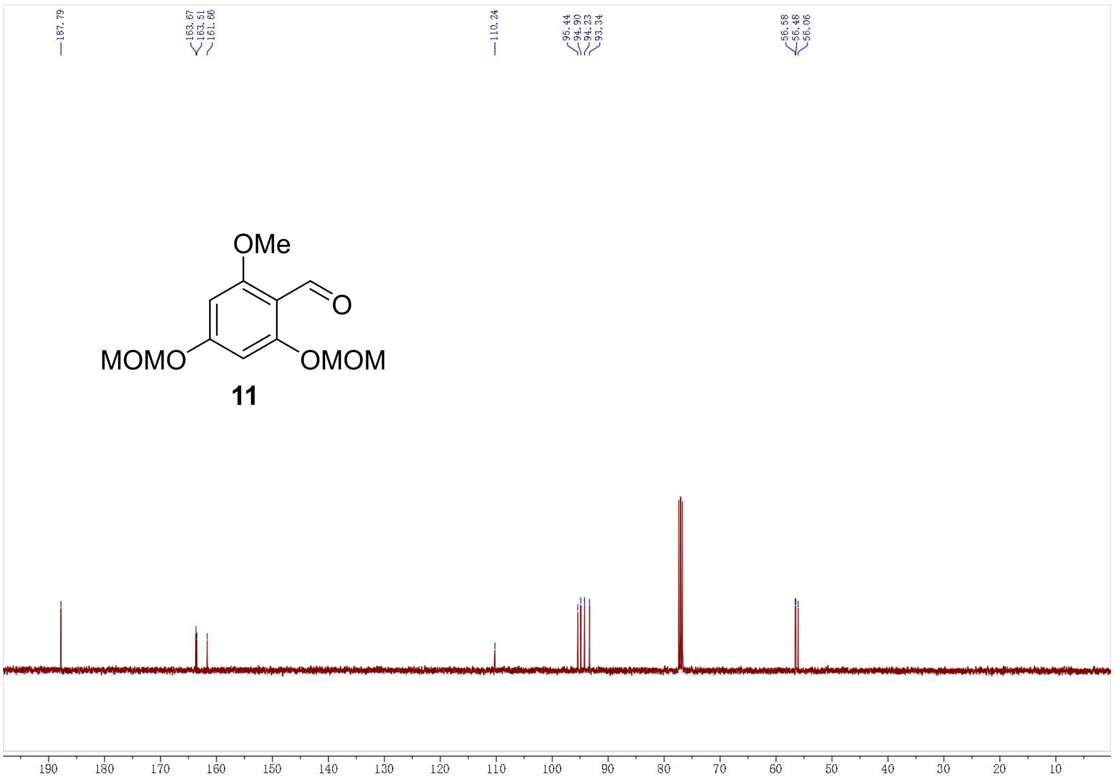

Figure S6 ^{13}C NMR (100 MHz, $CDCl_3$) of compound **11**

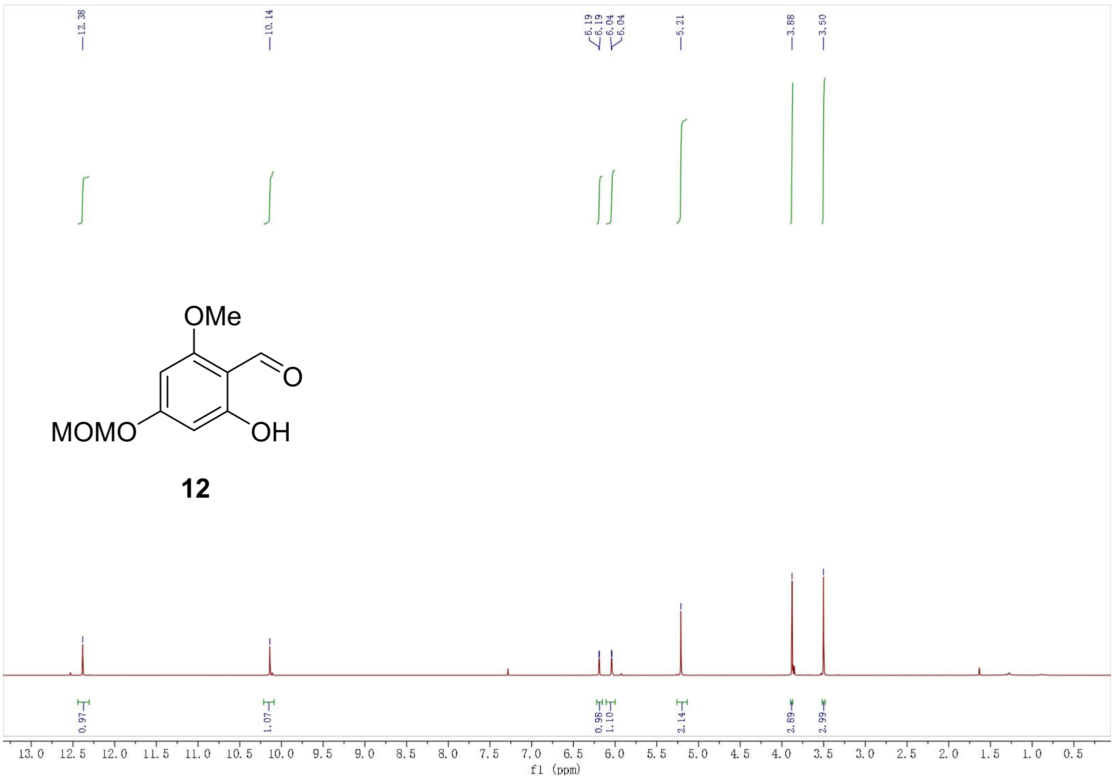

Figure S7 ^{1}H NMR (400 MHz, $CDCl_3$) of compound **12**

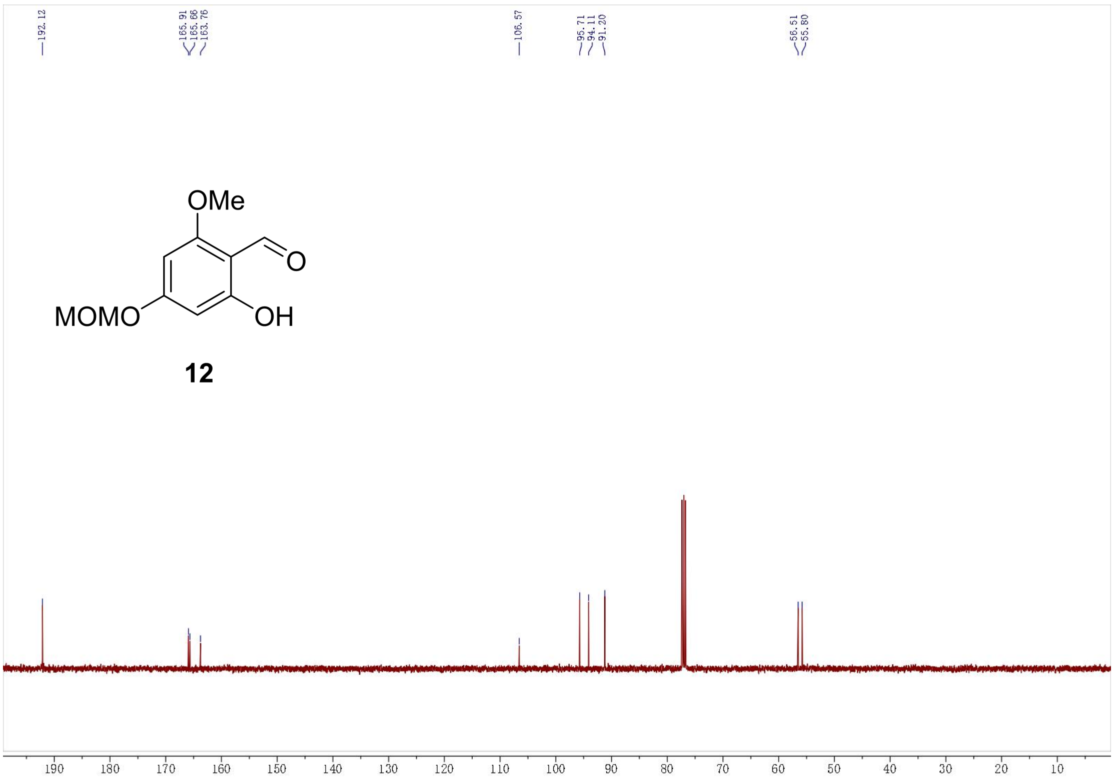

Figure S8 ^{13}C NMR (100 MHz, $CDCl_3$) of compound **12**

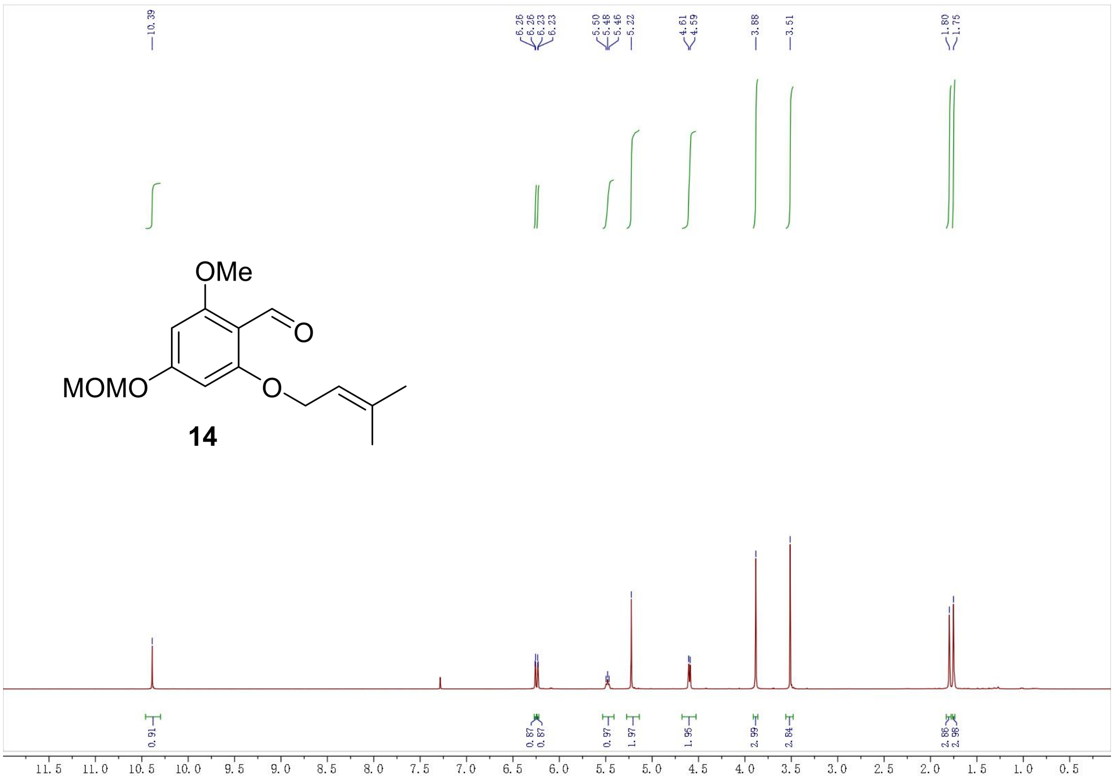

Figure S9 ^{1}H NMR (400 MHz, $CDCl_3$) of compound **14**

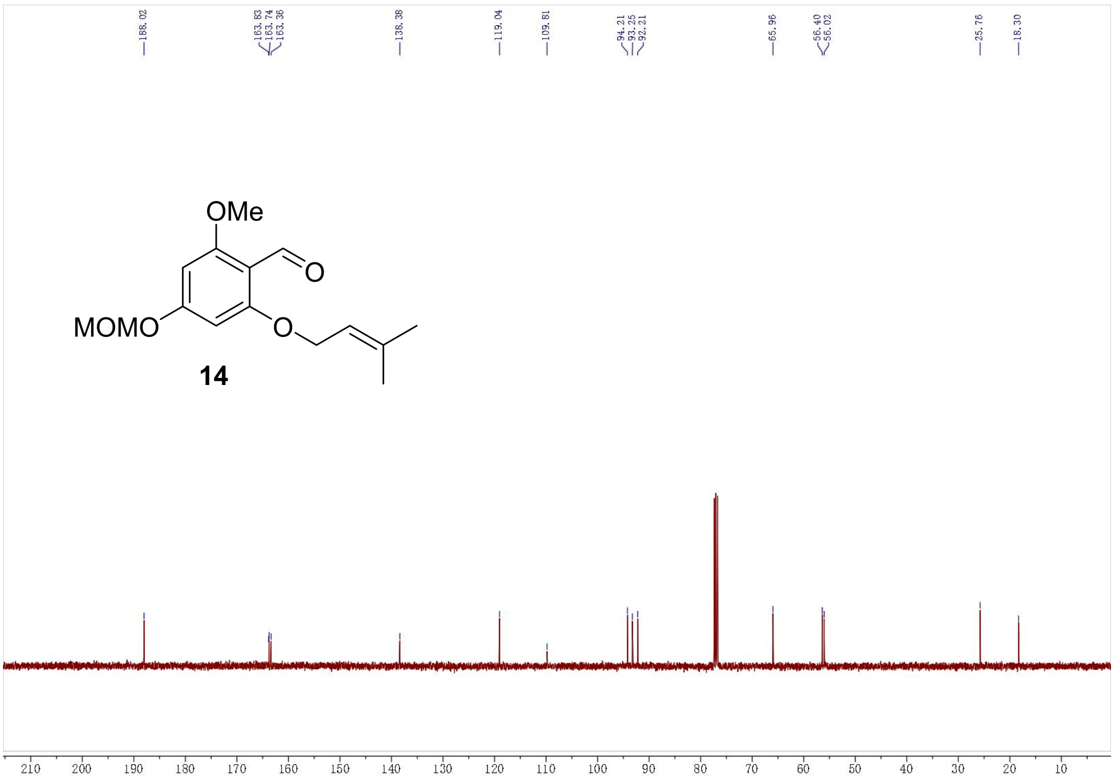

Figure S10 ^{13}C NMR (100 MHz, $CDCl_3$) of compound **14**

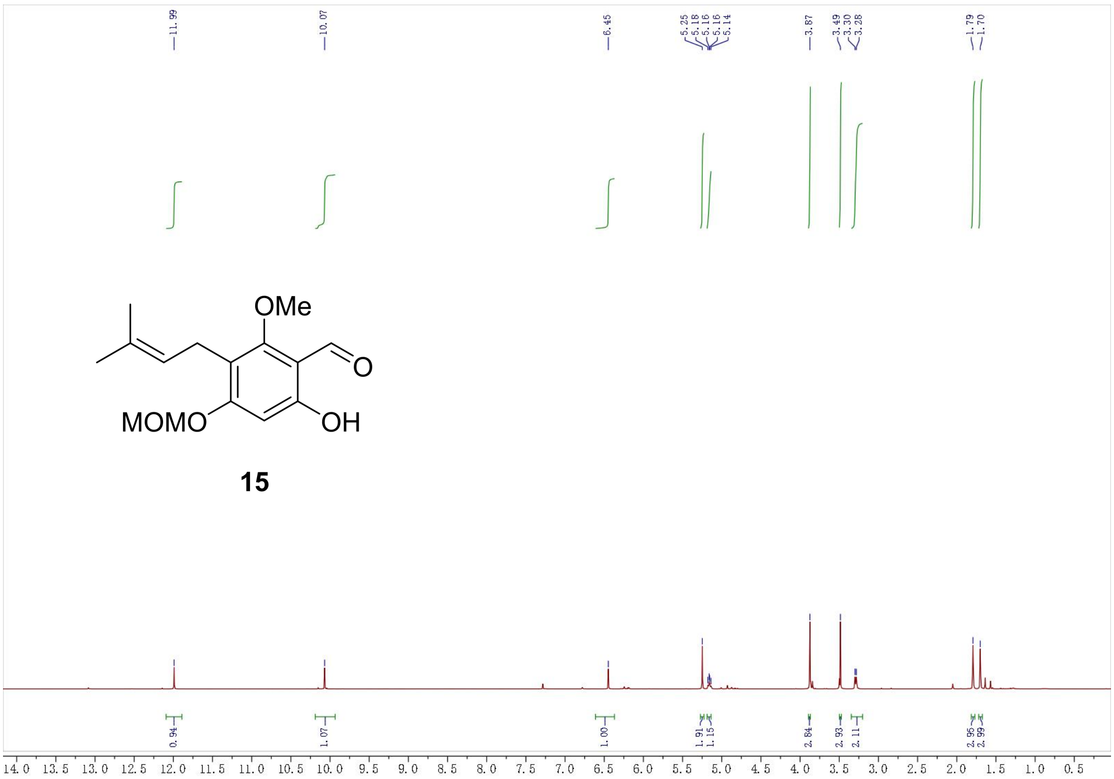

Figure S11 ^{1}H NMR (400 MHz, $CDCl_3$) of compound **15**

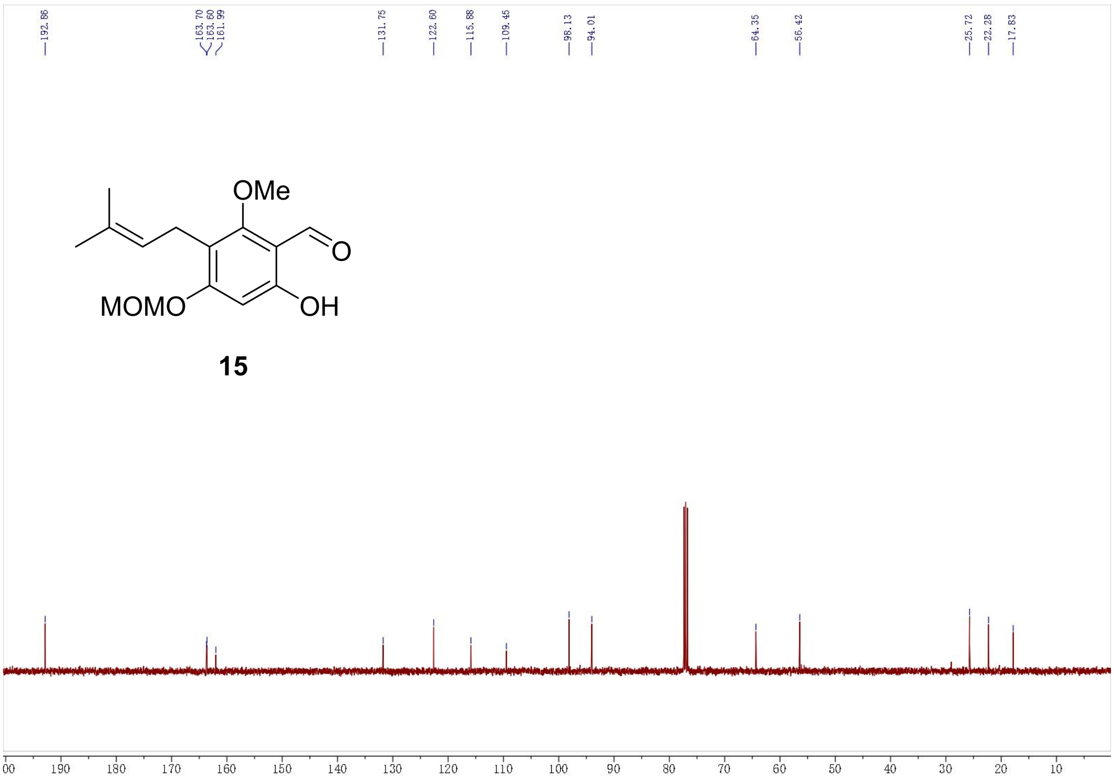

Figure S12 ^{13}C NMR (100 MHz, $CDCl_3$) of compound **15**

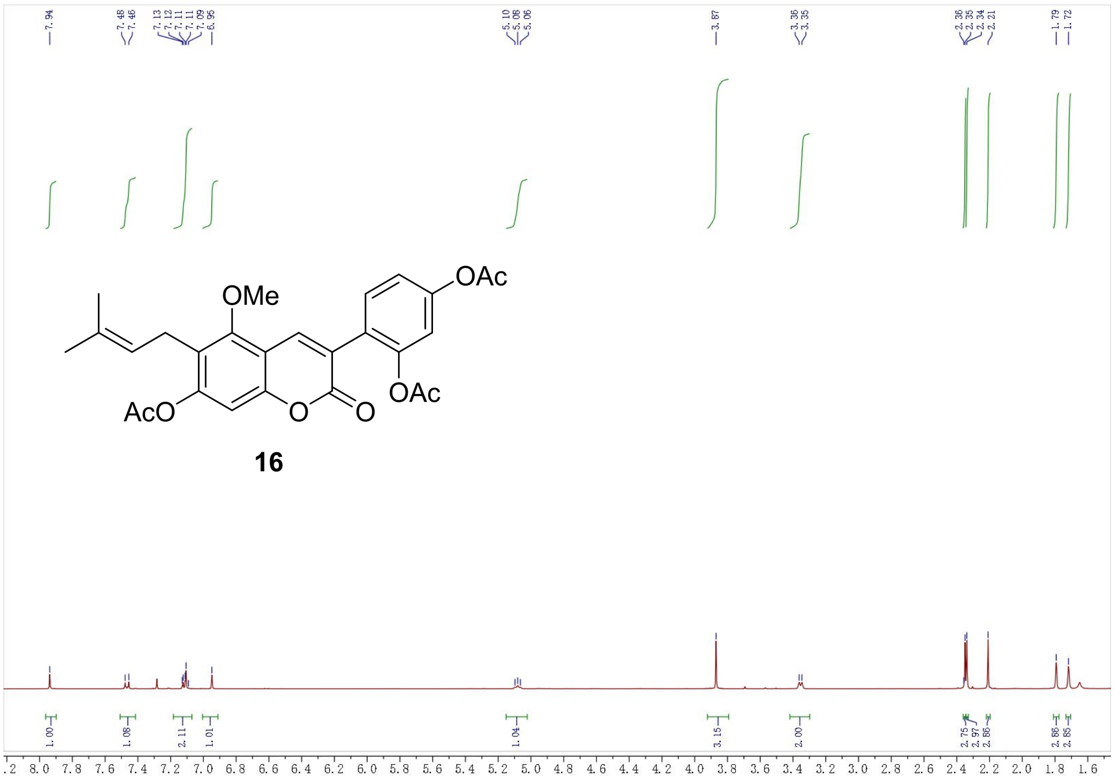

Figure S13 ^{1}H NMR (400 MHz, $CDCl_3$) of compound **16**

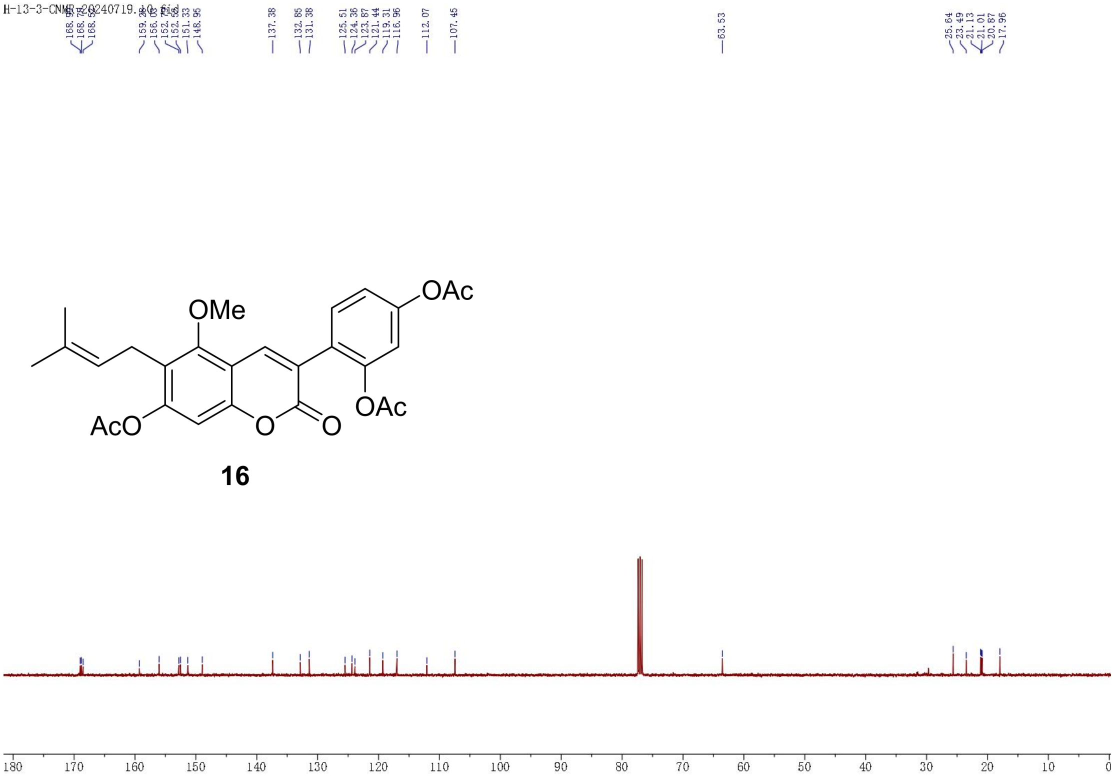

Figure S14 ^{13}C NMR (100 MHz, $CDCl_3$) of compound **16**

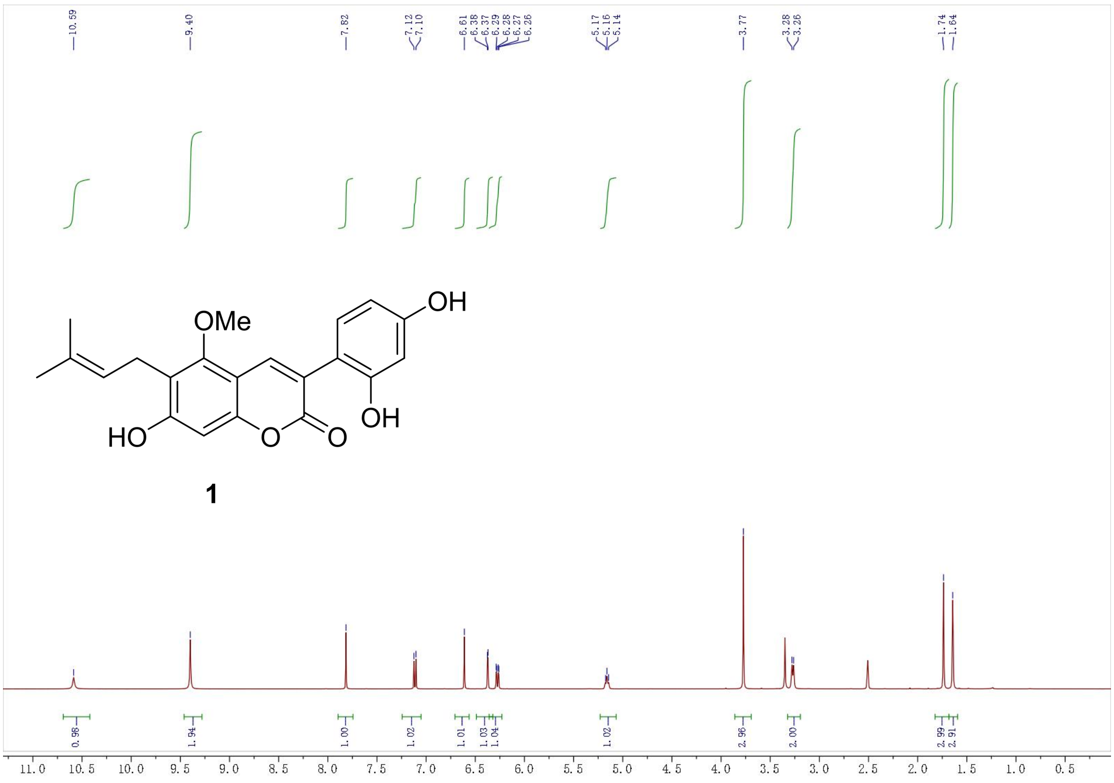

Figure S15 ^{1}H NMR (400 MHz, DMSO-d_6) of compound **1**

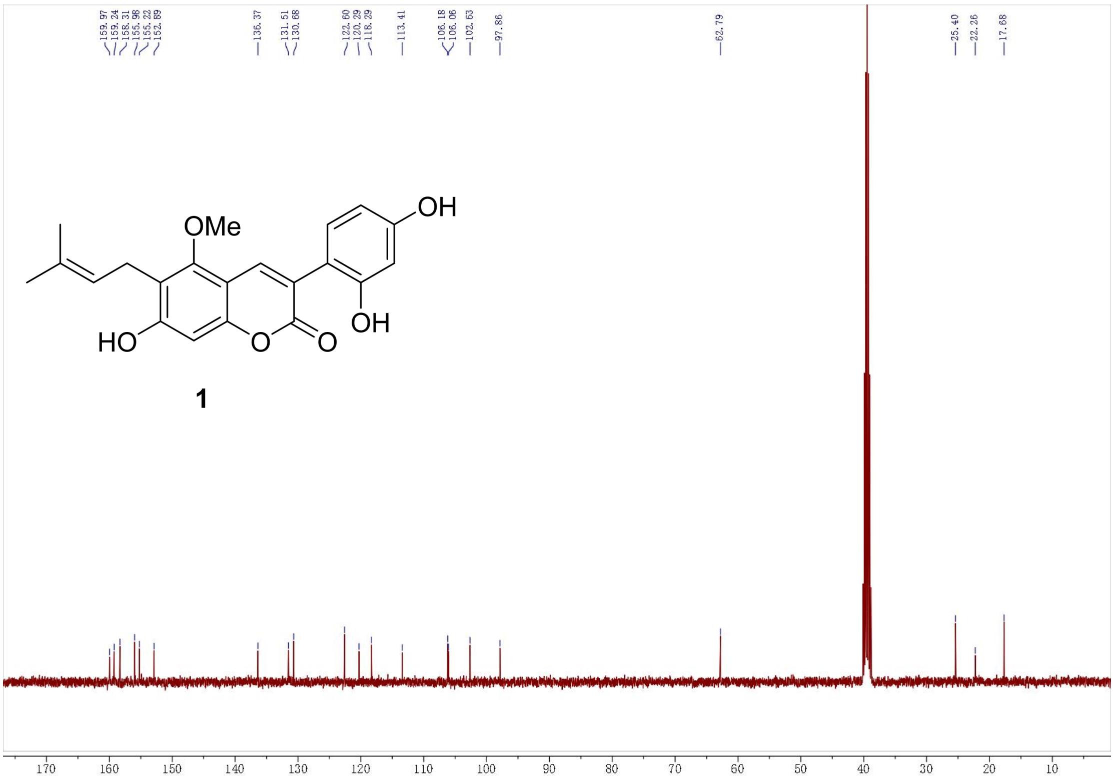

Figure S16 ^{13}C NMR (100 MHz, DMSO-d_6) of compound **1**

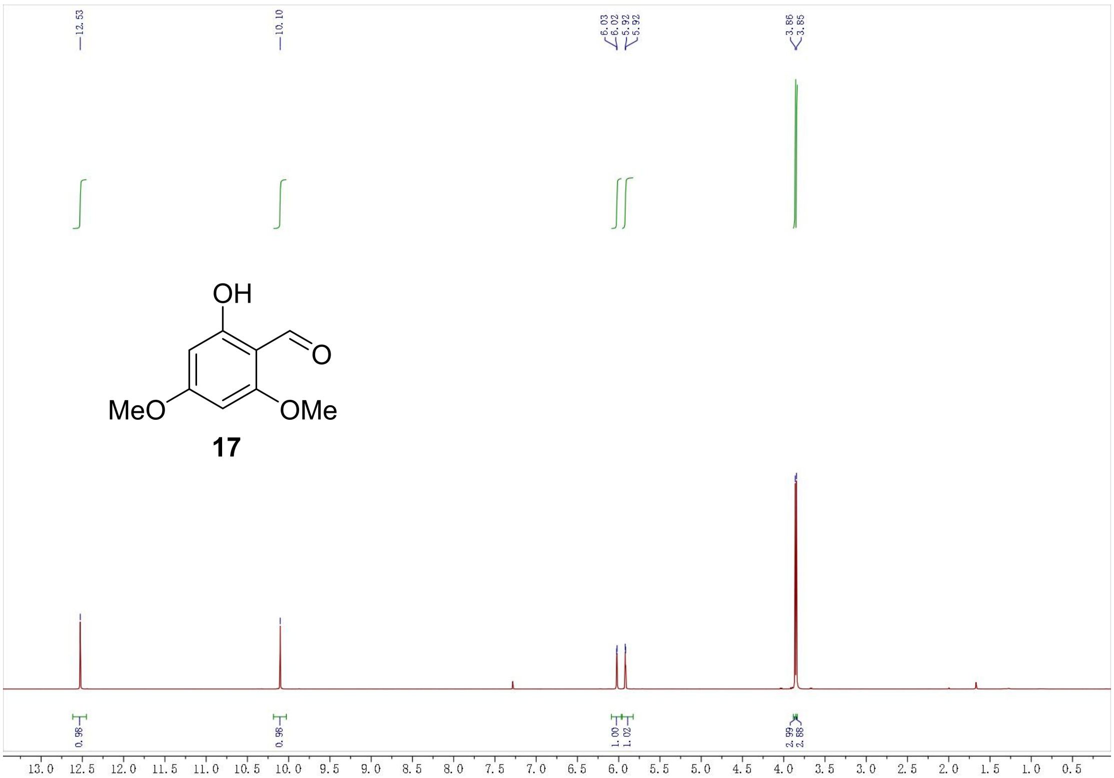

Figure S17 ^{1}H NMR (400 MHz, $CDCl_3$) of compound **17**

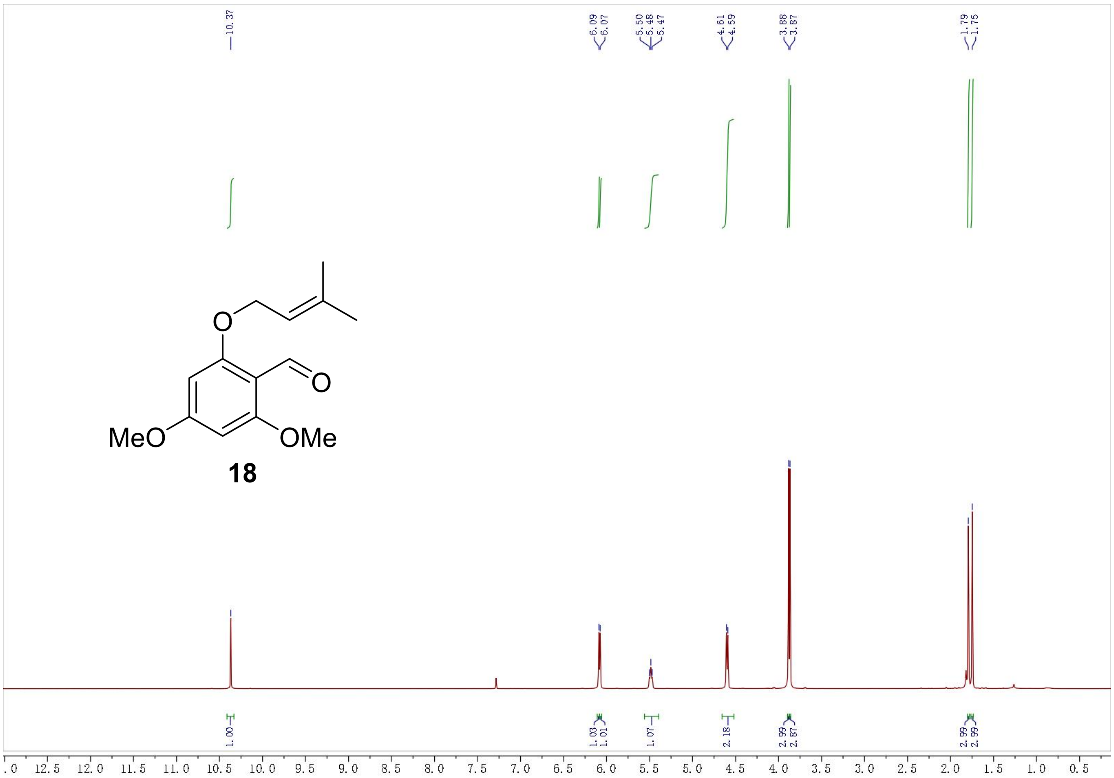

Figure S18 ^{1}H NMR (400 MHz, $CDCl_3$) of compound **18**

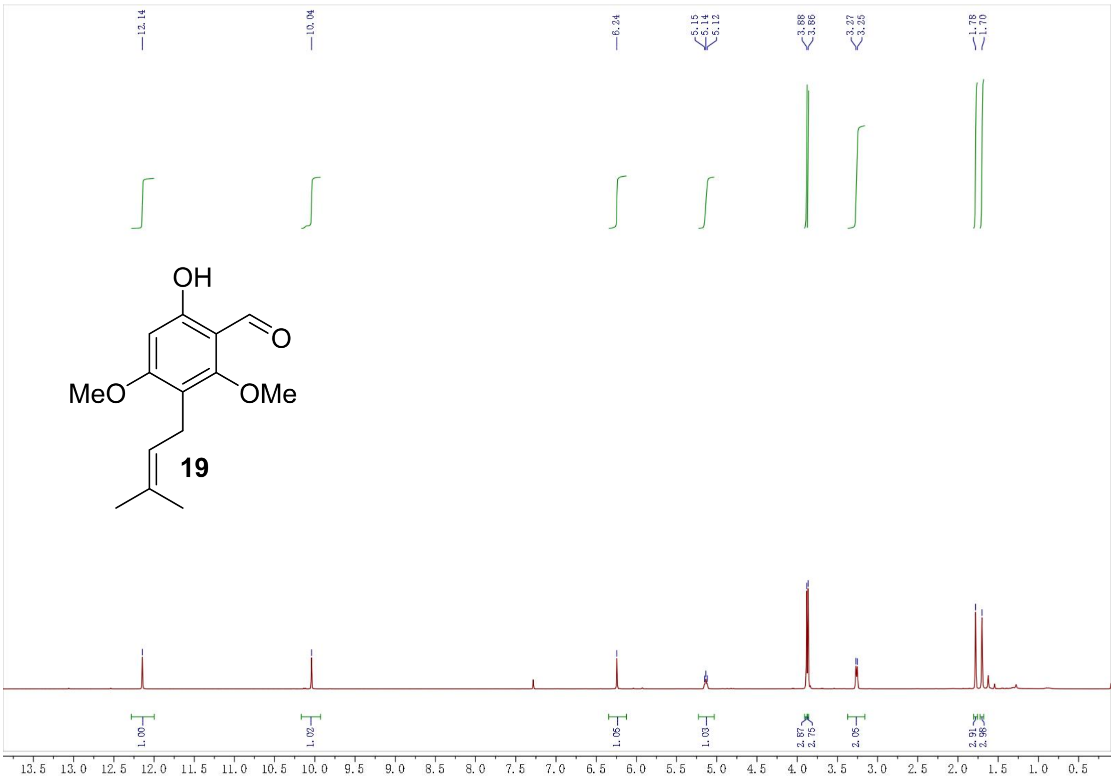

Figure S19 ^{1}H NMR (400 MHz, $CDCl_3$) of compound **19**

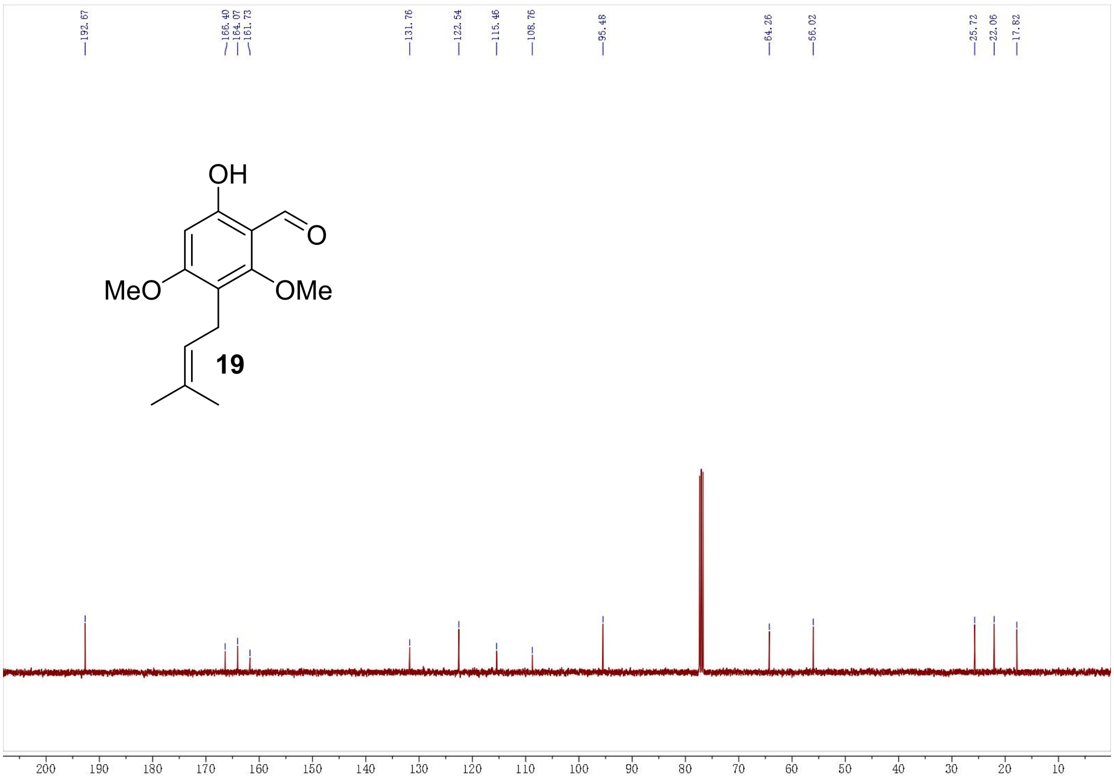

Figure S20 ^{13}C NMR (100 MHz, $CDCl_3$) of compound **19**

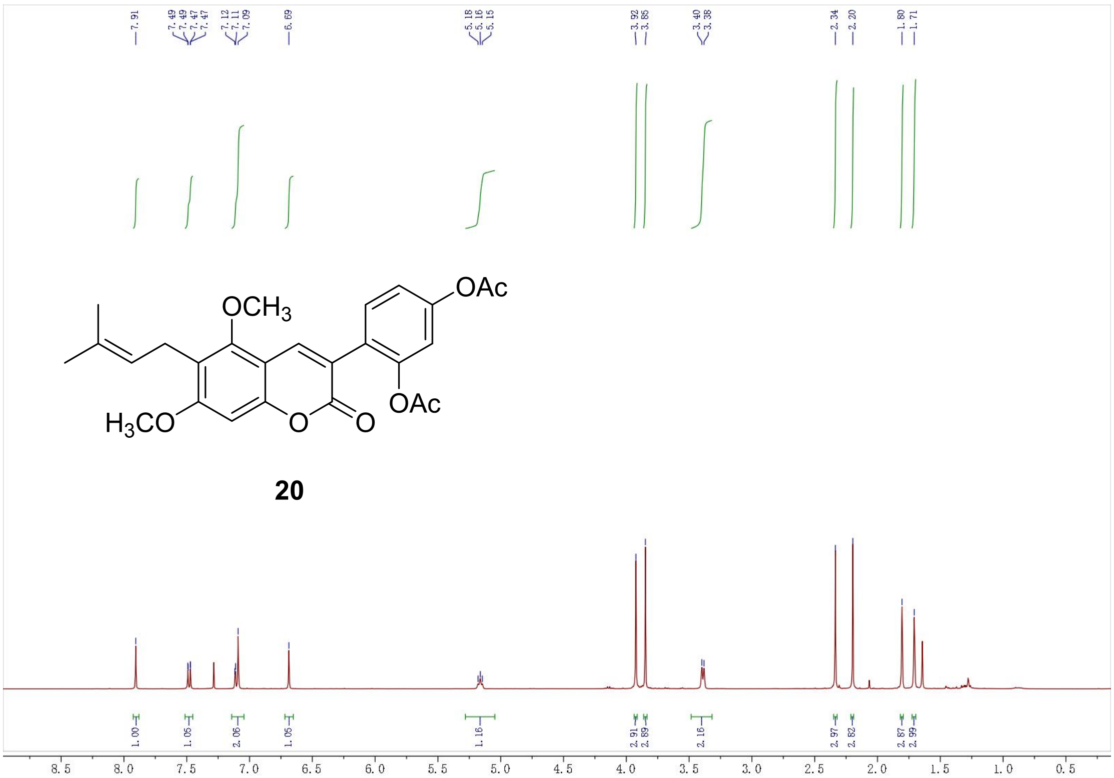

Figure S21 ^{1}H NMR (400 MHz, $CDCl_3$) of compound **20**

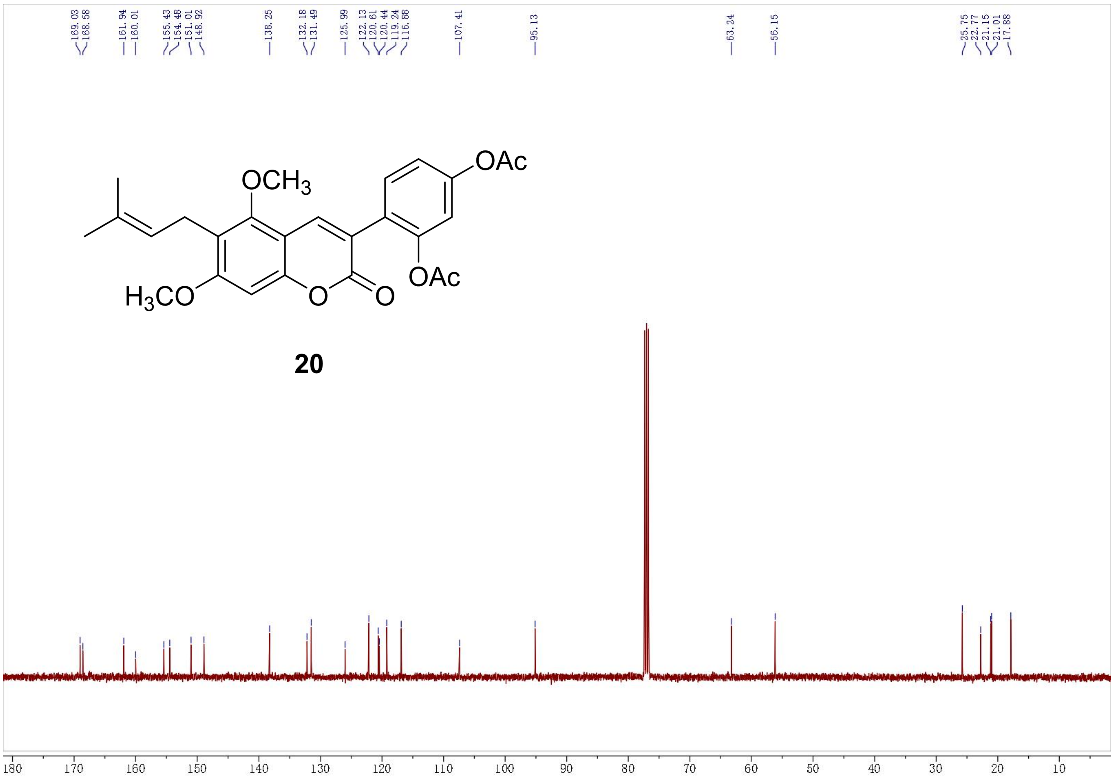

Figure S22 ^{13}C NMR (100 MHz, $CDCl_3$) of compound **20**

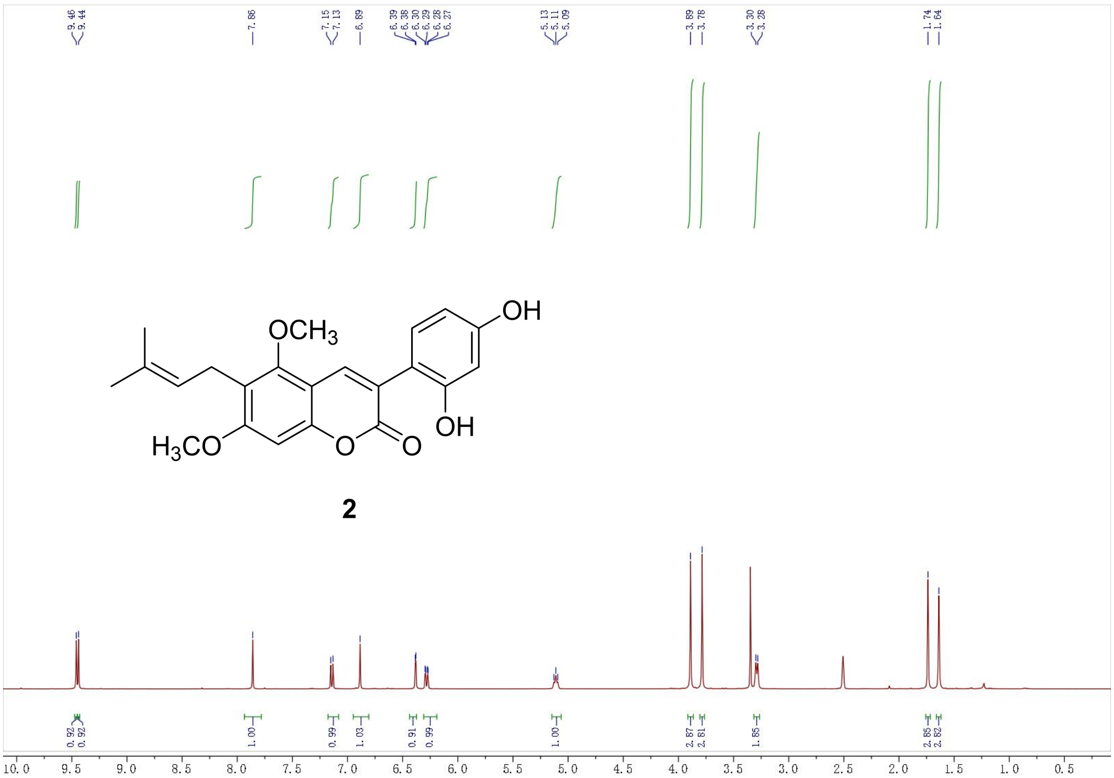

Figure S23 ^{1}H NMR (400 MHz, DMSO-d_6) of compound **2**

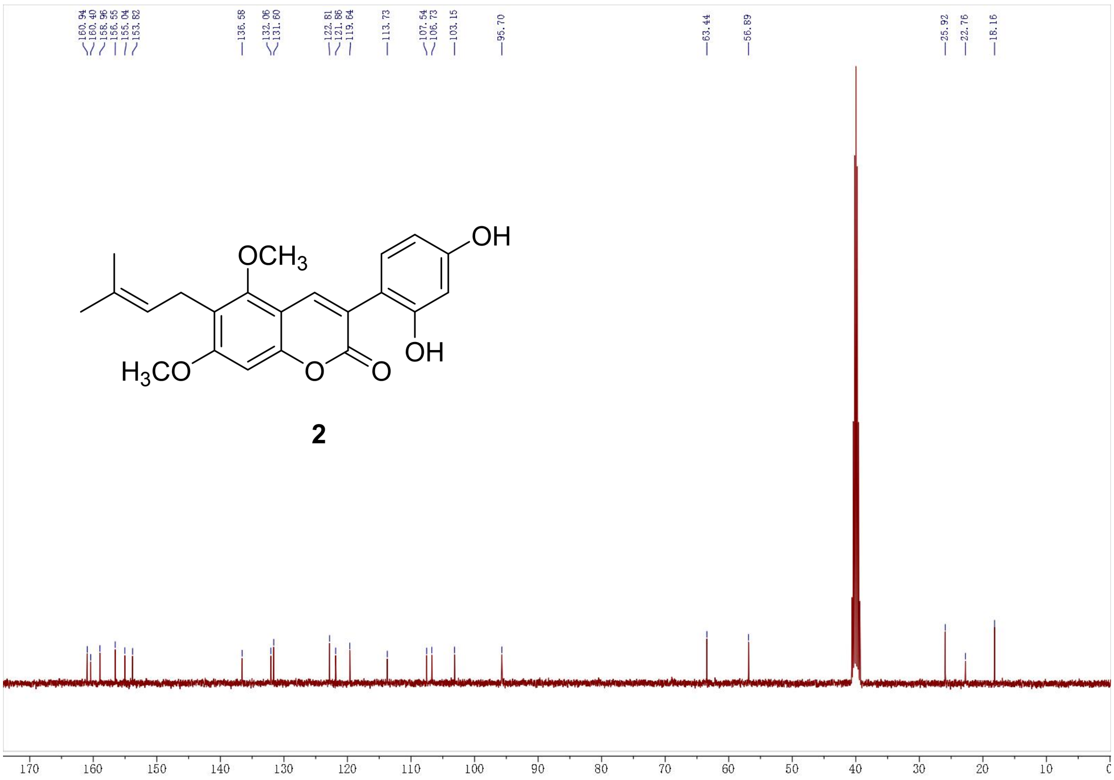

Figure S24 ^{13}C NMR (100 MHz, DMSO-d_6) of compound **2**

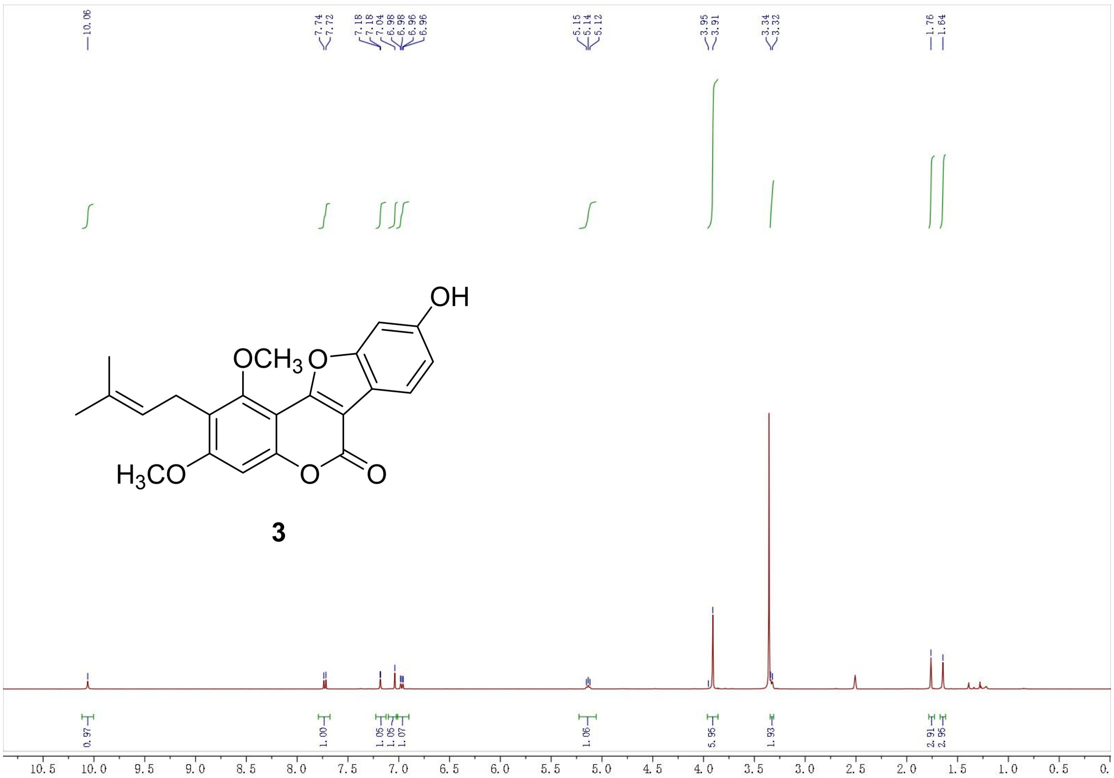

Figure S25 ^{1}H NMR (400 MHz, DMSO-d_6) of compound **3**

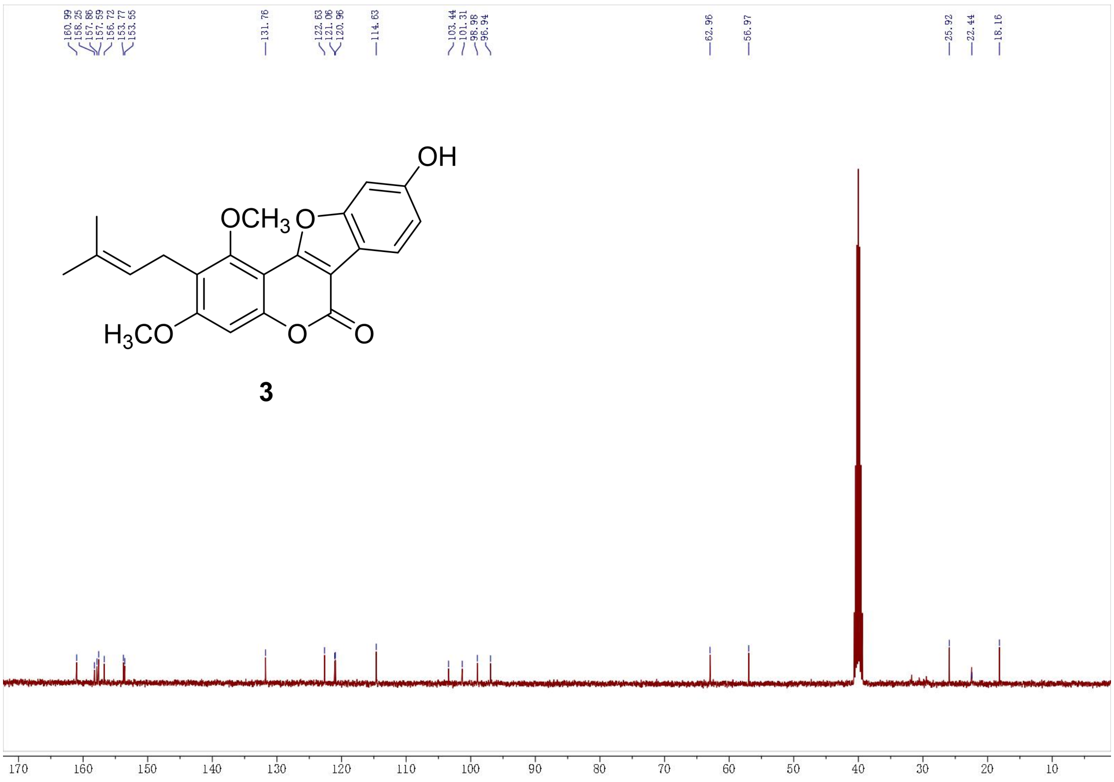

Figure S26 ^{13}C NMR (100 MHz, DMSO-d_6) of compound **3**
